# Gut AstA mediates sleep deprivation-induced energy wasting in *Drosophila*

**DOI:** 10.1038/s41421-023-00541-3

**Published:** 2023-05-23

**Authors:** Yingge Li, Xiaoya Zhou, Chen Cheng, Guangming Ding, Peng Zhao, Kai Tan, Lixia Chen, Norbert Perrimon, Jan A. Veenstra, Luoying Zhang, Wei Song

**Affiliations:** 1 Department of Hepatobiliary and Pancreatic Surgery, Medical Research Institute, Frontier Science Center of Immunology and Metabolism, Zhongnan Hospital of Wuhan University, Wuhan University, Wuhan, Hubei China; 2 TaiKang Center for Life and Medical Sciences, Wuhan University, Wuhan, Hubei China; 3 Key Laboratory of Molecular Biophysics of Ministry of Education, Hubei Bioinformatics and Molecular Imaging Key Laboratory, Center for Artificial Intelligence Biology, College of Life Science and Technology, Huazhong University of Science and Technology, Wuhan, Hubei China; 4 Department of Genetics, Howard Hughes Medical Institute, Harvard Medical School, Boston, MA USA; 5 INCIA, UMR 5287 CNRS, University of Bordeaux, Talence, France

**Keywords:** Mechanisms of disease, Stress signalling

## Abstract

Severe sleep deprivation (SD) has been highly associated with systemic energy wasting, such as lipid loss and glycogen depletion. Despite immune dysregulation and neurotoxicity observed in SD animals, whether and how the gut-secreted hormones participate in SD-induced disruption of energy homeostasis remains largely unknown. Using *Drosophila* as a conserved model organism, we characterize that production of intestinal Allatostatin A (AstA), a major gut-peptide hormone, is robustly increased in adult flies bearing severe SD. Interestingly, the removal of AstA production in the gut using specific drivers significantly improves lipid loss and glycogen depletion in SD flies without affecting sleep homeostasis. We reveal the molecular mechanisms whereby gut AstA promotes the release of an adipokinetic hormone (Akh), an insulin counter-regulatory hormone functionally equivalent to mammalian glucagon, to mobilize systemic energy reserves by remotely targeting its receptor AstA-R2 in Akh-producing cells. Similar regulation of glucagon secretion and energy wasting by AstA/galanin is also observed in SD mice. Further, integrating single-cell RNA sequencing and genetic validation, we uncover that severe SD results in ROS accumulation in the gut to augment AstA production via TrpA1. Altogether, our results demonstrate the essential roles of the gut-peptide hormone AstA in mediating SD-associated energy wasting.

## Introduction

Sleep plays a fundamental role in maintaining systemic metabolic homeostasis in many species^1,2^. Severe sleep deprivation (SD) is established to be associated with increased energy expenditure, weight loss, and decline of carbo-lipid storage in mammals^2^. Many groups have uncovered the participation of immune-response impairment, neuronal disturbance, and neurotoxicity in the brain, as well as oxidative stress and local catabolism in individual peripheral tissues based on rodent models and clinical investigations^3–7^. However, despite the gained knowledge, whether and how secreted factors mediate signals between metabolic organs and promote systemic energy catabolism under SD remains largely unknown.

Recent studies indicate that severe SD causes deleterious ROS accumulation predominantly in the intestine and perturbs intestinal homeostasis in both *Drosophila* and mice^8^. The gastrointestinal tract is a key endocrinal organ that modulates systemic energy homeostasis in response to environmental and physiological cues across species. In mammals, enteroendocrine cells (EEs) secrete distinct peptide hormones like CCK, GLP-1, and ghrelin to target multiple metabolic tissues and orchestrate systemic carbohydrate and lipid metabolism^9^. However, mammalian physiological complexity and gene redundancy have complicated functional investigations in the intestine. *Drosophila* has emerged as an evolutionary conserved model with similar but simple anatomy and physiology of the intestine. Researchers using the *Drosophila* have addressed significant aspects of intestinal endocrinal impacts, including EE heterogeneities, stress and nutrient sensing of EEs, types and functions of peptide hormones derived from EEs and other cells, as well as intestinal endocrinal regulation in diverse disease models^10–15^.

Two major subtypes of EEs are present in the *Drosophila* intestinal epithelium, tachykinin (Tk) and allatostatin A/C (AstA/C) EEs, which produce more than 20 mature peptide hormones^16–18^. Many groups and ours have demonstrated the essential metabolic roles of gut-peptide hormones that are produced by Tk EEs, including Tk, Neuropeptide F (NPF), Bursicon (Burs), and Activin-β (Actβ) to target enterocytes, ovary, neuroendocrine cells, and fat body to modulate systemic carbo-lipid balance^19–22^. In contrast, the physiological functions of peptide hormones derived from the other subtype AstA/C EEs are not well understood. AstA is a major peptide hormone that is expressed in AstA/C EEs^23^, as well as in the nervous systems^24^. Recent studies using different *AstA-GAL4* lines and mutations in *AstA* to manipulate systemic AstA expression, revealed that brain-derived AstA affects sleep, feeding, as well as animal development, via activation of certain neurons^24–27^. Nevertheless, AstA expression in the gut was inevitably perturbed in those studies, and the physiological roles of EE-derived AstA was not characterized so far.

In this study, we employed specific drivers that target AstA EEs in the midgut and demonstrated that gut-derived AstA is essential for the depletion of carbohydrate and lipid storages caused by SD. The molecular mechanisms include that gut-derived AstA remotely promotes Akh release by targeting its receptor AstA-R2 in Akh-producing cells (APCs). Interestingly, we also confirmed similar regulation of glucagon release by AstA/galanin in SD mice. Moreover, we show that AstA EEs express TrpA1 to sense accumulated ROS in the gut under SD and enhance AstA production, leading to energy wasting.

## Results

### Severe sleep loss results in energy wasting

**Fig. 1:**
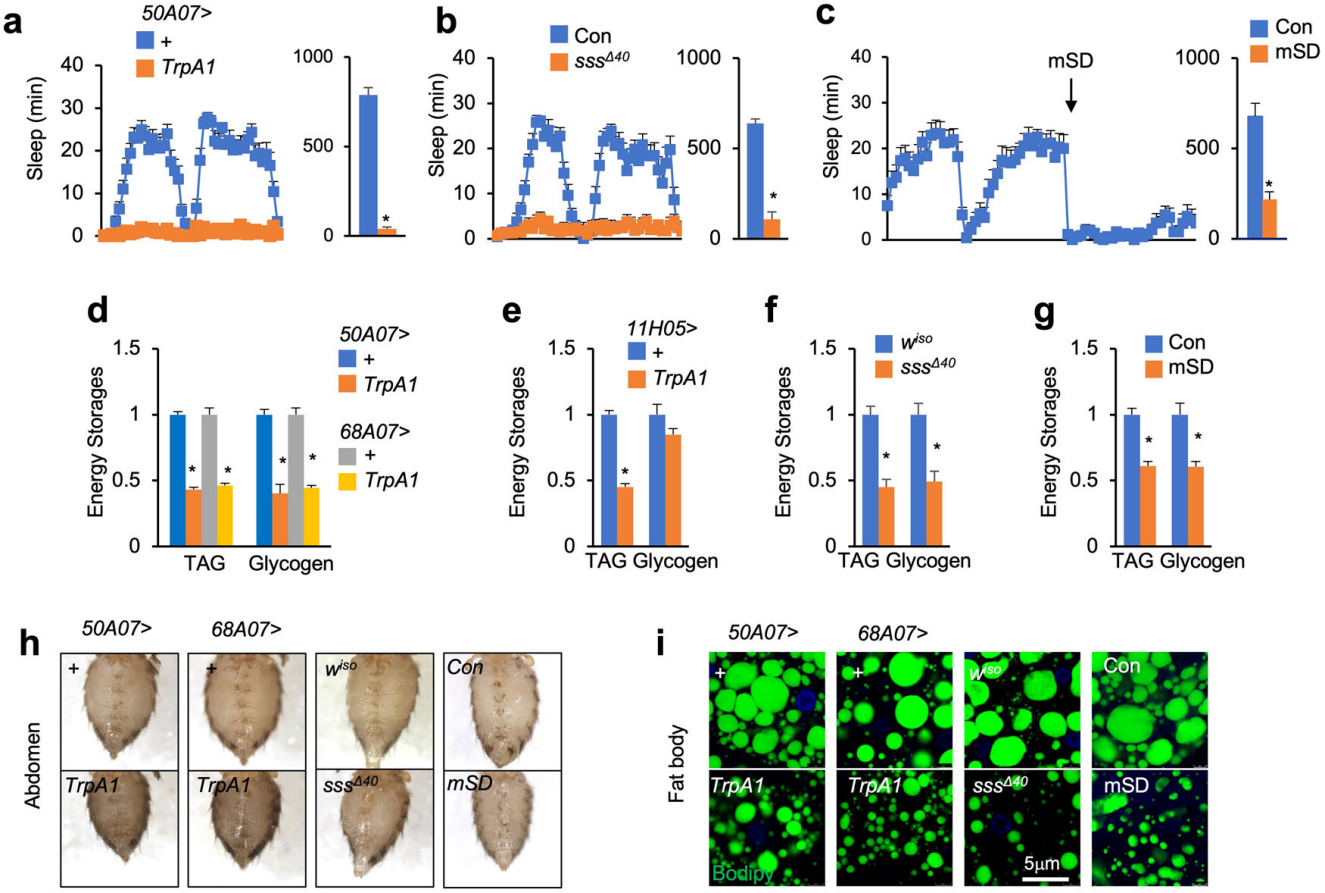
Severe SD causes energy wasting. **a**–**c** SD in 7-day-old *50A07* > *TrpA1* (*n* = 25) (**a**), *sss*^*Δ40*^ (*n* = 31) (**b**), mSD (*n* = 16) (**c**), and matched control female flies that were normally cultured (*n* = 20, left, sleep in every 30 min per day; right, total sleep per day). **d**–**g** TAG and glycogen storages of 7-day-old females of the indicated genotypes after SD for 10 days (**d**, **e**, TrpA1), 8 days (**f**, mutant), or 8 days (**g**, mechanical) (*n* = 4, 5 flies/replicate). **h**, **i** Abdomen appearance (**h**) and lipid droplets indicated by Bodipy staining (**i**, green) in abdominal fat bodies of the indicated genotypes after SD. Data were presented as means ± SEM. *P < 0.05.

Due to various degrees of sleep loss in different fly models, previous studies focusing on metabolic regulation under SD remain controversial^28–30^. To investigate whether severe SD impairs systemic energy homeostasis, we examined the levels of triglycerides (TAG) and glycogen, the major forms of stored lipids and carbohydrates, respectively, in adult flies with consistent sleep loss. We first performed thermogenetic stimulation by overexpressing heat-activated cation channel TrpA1 (TrpA1-A) to manually activate specific neurons at 29 °C using *50A07-LexA*, *68A07-LexA*, or *11H05-GAL4* drivers that had caused > 85% sleep loss^8^ (Fig. 1a). Interestingly, as compared to their control flies, both *50A07* > *TrpA1* and *68A07* > *TrpA1* flies exhibited a significant decrease in systemic TAG and glycogen levels after SD for 10 days (Fig. 1d and Supplementary Fig. S1h). *11H05* > *TrpA1* flies also showed a strong decrease in TAG storage but a mild decline in glycogen content (Fig. 1e). As a consequence of the TAG decrease, abdomens of *50A07* > *TrpA1* and *68A07* > *TrpA1* flies appeared more translucent with loss of the lipid layer (Fig. 1h). We examined the lipid content in the abdominal fat body using Bodipy staining and consistently observed a decreased mass of lipid droplets in *50A07* > *TrpA1* and *68A07* > *TrpA1* flies (Fig. 1i). We also examined *sleepless* (*sss*^*Δ40*^) mutant flies that showed > 85% sleep loss (Fig. 1b)^8,31,32^ and similarly observed loss of TAG and glycogen storages, translucent abdomens, as well as smaller lipid droplets, as compared to their control flies after SD for 8 days (Fig. 1f, h, i). However, another sleep-loss mutant flies, *DAT*^*fmn*^^33^, exhibited moderate sleep loss (< 50%) and no changes in both TAG and glycogen storages (Supplementary Fig. S1a, b). To further validate SD-induced metabolic imbalance, we examined flies that were mechanically deprived of sleep by ~80% using the vibration method as well (Fig. 1c)^8^. Consistently, flies with mechanical sleep deprivation (mSD) for 8 days exhibited loss of TAG and glycogen storages (Fig. 1g–i). These results collectively indicate that SD results in energy wasting in *Drosophila*.

### SD increases AstA production in the gut

**Fig. 2:**
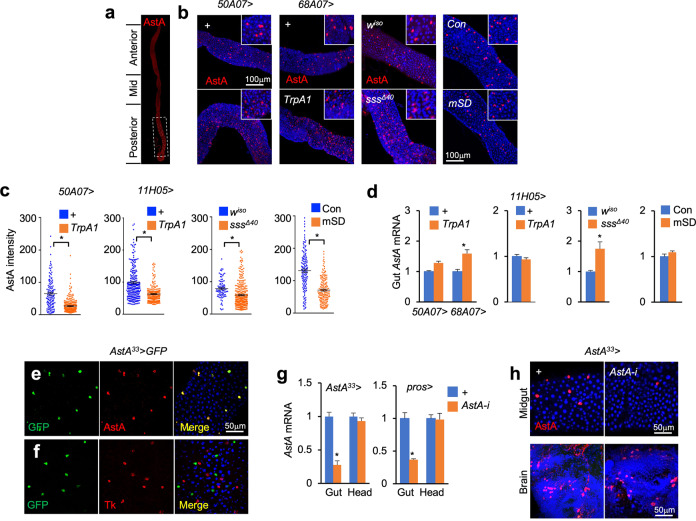
SD increases AstA production. **a** Distribution of AstA EEs as indicated by AstA immunostaining (red) in the posterior region of the midgut of 7-day-old WT flies. **b**–**d** Immunostaining of AstA (**b**, red), quantification of intracellular AstA signal in each single cell (**c**, *n* > 80), and *AstA* mRNA levels (**d**, *n* = 3, 15 flies/genotype) in the midguts of 7-day-old female flies after SD for 10 days (TrpA1), 8 days (mutant), or 8 days (mSD). **e**, **f** Immunostaining indicating intestinal expressions of AstA (**e**) and Tk (**f**) in 8-day-old AstA^33^ > GFP flies. **g**, **h** mRNA (**g**, *n* = 3, 15 flies/replicate) and protein (**h**, up, posterior midgut; bottom, superior medial protocerebrum of the brain) levels of AstA in the midguts and brains in indicated 7-day-old female flies. Data were presented as means ± SEM. *P < 0.05.

*Drosophila* gut-peptide hormones have been shown to extensively modulate systemic energy homeostasis^11^, we thus wonder whether SD modulates the production of certain gut-peptide hormones to execute the impacts on carbo-lipid metabolism. We examined a few known gut-peptide hormones and found robust changes in the production of gut AstA under SD. AstA was found to be majorly produced in the posterior region of the midgut (Fig. 2a). Importantly, intracellular AstA protein levels in the gut of these SD flies were potently decreased (Fig. 2b, c), while the gut *AstA* mRNA levels remained unchanged or slightly increased (Fig. 2d), in *50A07* > *TrpA1*, *68A07* > *TrpA1*, *sss*^*Δ40*^, and mSD flies, indicating enhanced AstA release. We noticed an increase in the numbers of gut AstA EEs as well (Fig. 2b). By contrast, *DAT*^*fmn*^ flies with moderate sleep loss exhibited no changes in gut AstA production (Supplementary Fig. S1c, d).

We next investigated whether gut AstA production is essential for energy wasting-associated SD by manipulating AstA expression specifically in the gut. Similar to the previous characterization of *Tk-gut-GAL4* that predominantly targets Tk EEs in the gut^19^, we screened multiple *AstA-GAL4* lines and managed to identify one, referred to as *AstA*^*33*^*-GAL4*, that is expressed in all AstA EEs as the other heterologous pair of EEs opposite to Tk in the posterior midgut (Fig. 2e, f) and in very few AstA neurons (two in the protocerebrum, several in the optic lobe, one in the ventral nerve cord (VNC), but not in gnathal ganglia (GNG) region) (Supplementary Fig. S2a, b). Because *pros-GAL4* is a well-established driver targeting all gut EEs in *Drosophila*^34^, we also examined it and found its expression in all AstA EEs of the gut but rarely in AstA neurons in both brain and VNC (Supplementary Fig. S2c, d). We next expressed double-stranded RNAs against *AstA* (RNAi) to knock down gut *AstA* expression under the control of *AstA*^*33*^*-* or *pros-GAL4*. As compared with control, *AstA* expression was only diminished in the midgut, not the brain, as indicated by both quantitive RT-PCR (qPCR) and immunostaining (Fig. 2g, h and Supplementary Fig. S3a, b).

### Gut AstA is required for SD-induced energy wasting

**Fig. 3:**
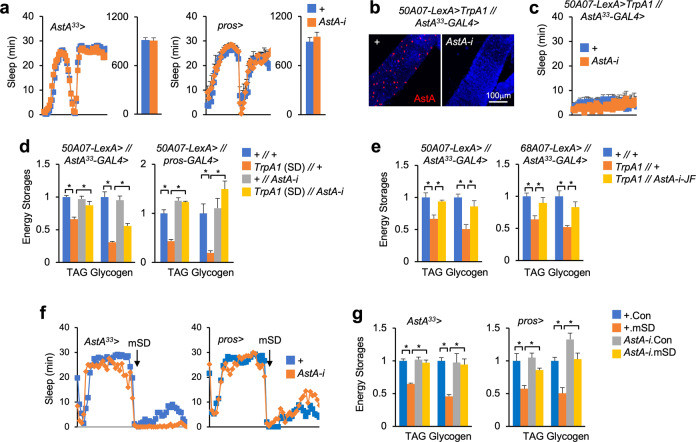
Gut AstA deficiency restores energy balance in SD flies. **a** Sleep of 6-day-old female flies with indicated genotypes (*n* = 31 for *AstA*^*33*^>, *n* = 16 for *pros*>, left, sleep in every 30 min per day; right, total sleep per day). **b** Immunostaining of intestinal AstA (red) in flies of indicated genotypes after SD for 10 days. **c** Sleep in every 30 min per day of indicated flies (+, *n* = 30; *AstA-i*, *n* = 25). **d**, **e** TAG and glycogen storages of 7-day-old females of the indicated genotypes with or without SD for 10 days (*n* = 4, 5 flies/replicate). **f** Sleep of 5-day-old female flies with indicated genotypes (*n* = 15 for *AstA*^*33*^>, *n* = 16 for *pros*>, left, sleep in every 30 min per 24/48 h). **g** TAG and glycogen storages of 5-day-old females of the indicated genotypes with or without mSD for 8 days (*n* = 4, 5 flies/replicate). Data were presented as means ± SEM. *P < 0.05.

These genetic tools allowed us to manipulate AstA expression specifically in the gut and investigate its metabolic roles in SD-associated energy wasting. First, we found that gut *AstA* knockdown in control flies driven by either *AstA*^*33*^*-* or *pros-GAL4* did not affect sleep homeostasis (Fig. 3a). In *50A07* > *TrpA1* SD flies, gut AstA deficiency did not improve severe sleep loss either (Fig. 3b, c), excluding the impacts of gut AstA on sleep regulation. We next examined the metabolic changes and found that gut AstA removal using either *AstA*^*33*^*-* or *pros-GAL4* in control files with normal sleep hardly affected the systemic storages of TAG and glycogen (Fig. 3d). Strikingly, gut AstA deficiency dramatically improved the loss of TAG and glycogen reserves in *50A07* > *TrpA1* flies and restored them to the levels close to control flies (Fig. 3d). We further confirmed these results using another independent *AstA* RNAi line as well as *68A07* > *TrpA1* SD flies (Fig. 3e). Note that, we hardly observed the expression of *50A07-LexA*, *68A07-LexA* and *11H05-GAL4* in either AstA EEs or neurons (Supplementary Fig. S3a–d). Further, flies bearing only *UAS-TrpA1, UAS-AstA-RNAi*, or *LexAop-TrpA1* insertion exhibited normal sleep and carbo-lipid metabolism (Supplementary Fig. S1f–h). These results excluded the possibilities that leaky expression of TrpA1 in AstA neurons leads to energy wasting. Finally, we consistently observed that, in flies with mSD, gut AstA deficiency using either *AstA*^*33*^*-* or *pros-GAL4* potently rescued the loss of TAG and glycogen reserves without affecting sleep loss (Fig. 3f, g). By contrast, the knockdown of *AstA* in the brain by > 50%, but not in the gut, using a weak strain of *elav-GAL4* failed to affect energy wasting in mSD flies (Supplementary Fig. S1i–k). Taken together, our results demonstrate that gut AstA production is essential for SD-induced energy wasting.

### Gut AstA remotely promotes Akh release

Since it is well established that nutrients regulate the production of gut-peptide hormones, we also investigated the metabolic roles of gut AstA under nutrient availability. Similar to SD, starvation for 36 h significantly increased gut AstA release as indicated by the decline of intracellular AstA protein (Supplementary Fig. S4a) and systemic storages of TAG and glycogen (Supplementary Fig. S4b). Interestingly, gut AstA deficiency using two different RNA lines (Supplementary Fig. S4b–d), as well as Rpr-induced AstA-EE ablation (Supplementary Fig. S4e–h), potently restored systemic TAG and glycogen storages after starvation and increased fly survival rates during starvation.

We asked how AstA modulates systemic metabolic homeostasis and exploited the potential molecular mechanisms. Akh is a metabolic hormone that is released from APCs upon nutrient deprivation to activate AkhR/cAMP/Ca^2+^ cascade in the fat body, leading to the carbo-lipid utilization by promoting target gene expression associated with lipolysis and glycogenolysis^35–38^. As the metabolic phenotypes associated with gut AstA deficiency are reminiscent of the phenotype of Akh mutants, we tested whether gut AstA regulates carbo-lipid metabolism through Akh. To monitor Akh release, we generated a polyclonal antibody from a rabbit against *Drosophila* Akh (see Materials and Methods). The immunostaining results indicated that the signals of the Akh antibody overlap with APCs only in control but not Akh-null mutant flies (*Akh*^*A*^ and *Akh*^*SAP*^)^39^ (Supplementary Fig. S5a, b). No obvious signals of Akh antibody were found in the brain as well, indicating no interactions between our Akh antibody and Wamide, Akh-related, or other peptides (Supplementary Fig. S5c). We also observed increased Akh amounts in the hemolymph of control but not Akh-null mutant flies under starvation using dot-blot assays (Supplementary Fig. S5d), demonstrating the specificity of our Akh antibody. Interestingly, gut AstA removal robustly suppressed Akh release, as indicated by decreased Akh amounts in the hemolymph and increased intracellular Akh accumulation in APCs (Supplementary Fig. S5e, f), and suppressed systemic expression of Akh-target gene *tobi* (Supplementary Fig. S6g) under starvation. These results indicate that gut AstA remotely promotes Akh release.

AstA-R2, a GPCR of AstA, has been previously reported to be expressed in the APCs^25^. We confirmed this result using a specific *AstA-R2-GAL4* driver that expresses GFP in all endogenous AstA-R2-expressing cells (Supplementary Fig. S6a). Consistent with gut *AstA* deficiency, knockdown of *AstA-R2* expression in APCs using *Akh-GAL4* led to a lower intracellular Ca^2+^ level as indicated by CaLexA reporter^40^, increased intracellular Akh accumulation, reduced *tobi* expression, as well as elevated TAG and glycogen storages, under starvation and decreased starvation-induced mortality (Supplementary Fig. S6b–d, g, h).

To investigate whether the metabolic actions of AstA is Akh dependent, we tested the effect of gut AstA removal in *Akh*^*A/SAP*^ mutant flies. Similar to the effect of loss of gut AstA, TAG storages after starvation were increased by ~50% in *Akh*^*A/SAP*^ flies (Supplementary Fig. S6e). Strikingly, gut AstA knockdown using *AstA*^*33*^*-GAL4* in *Akh*^*A/SAP*^ flies did not cause a further increase in TAG levels as compared to either *Akh*^*A/SAP*^ or *AstA*^*33*^ > *AstA-i* alone (Supplementary Fig. S6e). We also simultaneously knocked down *AstA* in the midgut and *AstA-R2* in the APCs using *AstA*^*33*^*-GAL4* and *Akh-GAL4*, respectively. Again, TAG storages and *tobi* expression after starvation, as well as starvation resistance, were not further increased by gut *AstA* deficiency plus *AstA-R2* knockdown in APCs (Supplementary Fig. S6f–h). Altogether, these results demonstrate that gut AstA regulates metabolic homeostasis by remotely enhancing Akh release.

### SD augments Akh release via gut AstA production

**Fig. 4:**
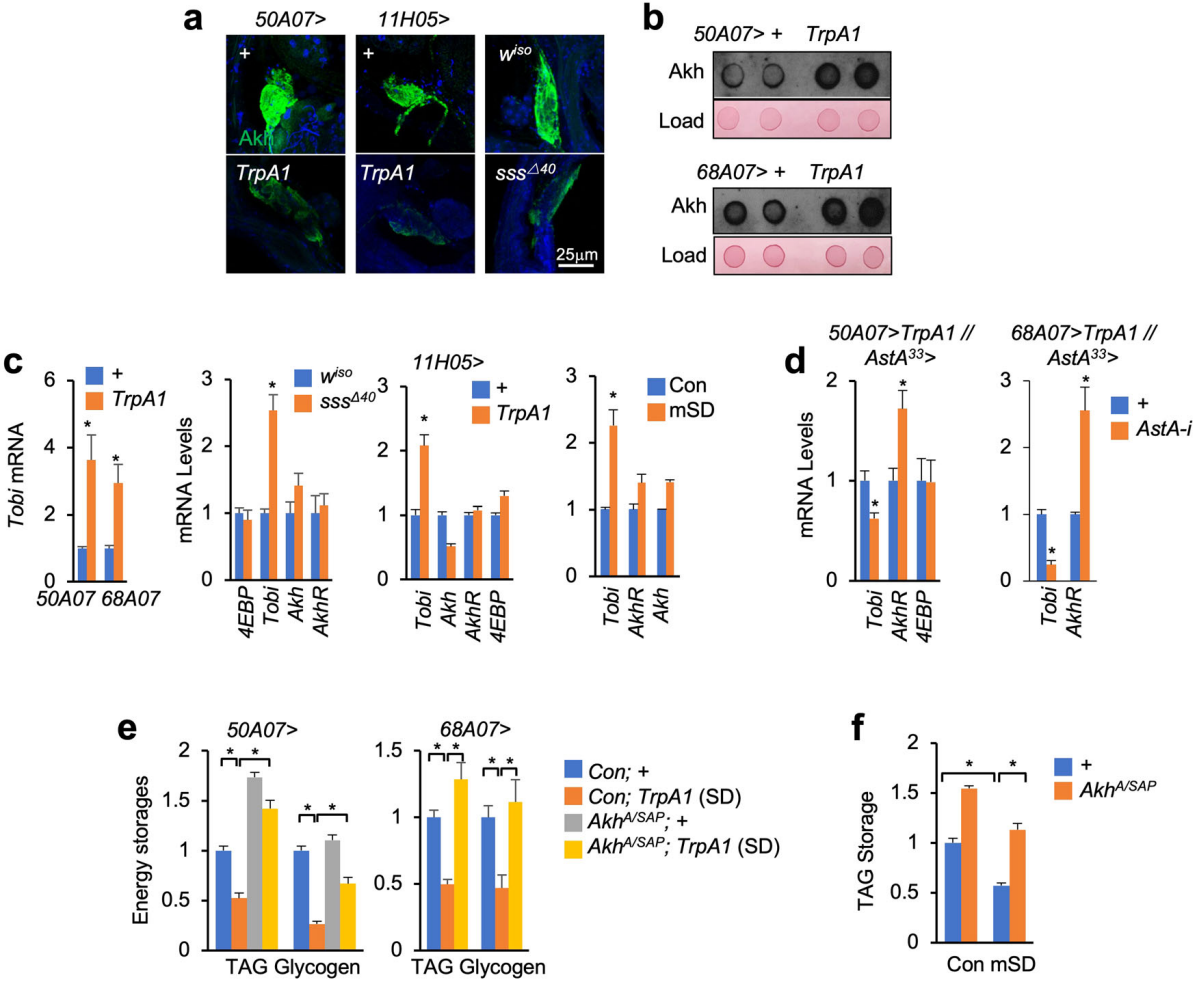
Gut AstA promotes Akh release to mediate SD-induced energy wasting. **a**, **b** Confocal images (*Z*-stack projection) of intracellular Akh (**a**, green) and hemolymph Akh amounts (**b**, loading protein amounts were indicated by Ponceau S staining) of the indicated 6-day-old flies after SD for 10 days (TrpA1) or 8 days (mutant). **c**–**f** Whole-body gene expression (**c**, **d**, *n* = 4, 5 flies/replicate) and TAG and glycogen storages (**e**, **f**, *n* = 4, 5 flies/replicate) of indicated flies after SD for 10 days (TrpA1), 8 days (mutant), or 8 days (mSD). Data were presented as means ± SEM. *P < 0.05.

We wonder whether AstA–Akh axis contributes to SD-induced energy wasting. To address this hypothesis, we first examined the Akh release under SD. Interestingly, decreased intracellular Akh accumulation in APCs, elevated circulating Akh amounts in the hemolymph, and increased *tobi* expression were consistently observed in different SD flies (Fig. 4a–c), indicating enhancement of Akh secretion and systemic Akh response. Note that, we failed to observe any changes in systemic expression of *4EBP*, an indicator of insulin signaling, in SD flies (Fig. 4c). We next knocked down gut *AstA* expression in SD flies and, as expected, observed suppression of systemic Akh responses as indicated by *tobi* expression (Fig. 4d). Furthermore, in order to examine whether Akh is essential for SD-induced energy wasting, we performed SD in *Akh*^*A/SAP*^ mutants using both thermogenetic and mechanical methods. We found that *Akh* deficiency potently restored TAG and glycogen storages in *50A07* > *TrpA1*, *68A07* > *TrpA1*, and mSD flies (Fig. 4e, f). Even though *Akh* deficiency elevated TAG and glycogen storages at basal conditions (Fig. 4e, f), we still observed that both thermogenetic and mSD resulted in TAG and glycogen loss to a much lesser extent in *Akh*^*A/SAP*^ mutant flies as compared to that in control flies (Fig. 4e, f). Collectively, our data demonstrate that SD augments Akh release via gut AstA production to cause energy wasting.

### Gut galanin promotes glucagon release to impair carbo-lipid metabolism in SD mice

**Fig. 5:**
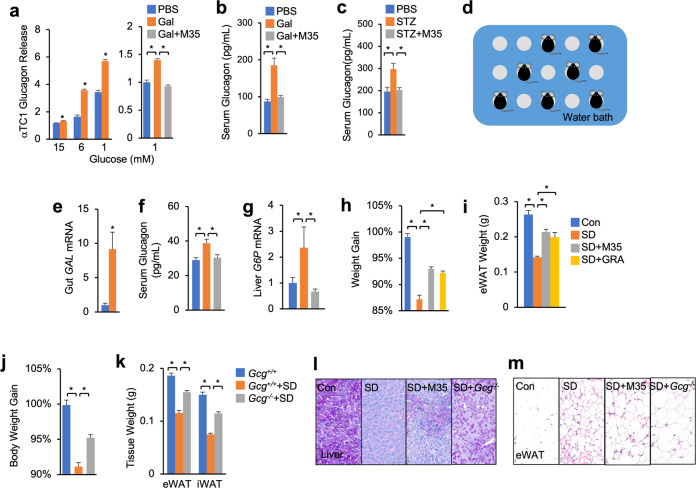
Galanin/glucagon axis mediates sleep loss-induced energy wasting in mice. **a** Glucagon secretion (R&D ELISA kit) in αTC1 cells that were treated with 100 nM galanin (Gal) or 1 nM M35 for 4 h in DMEM containing different doses of glucose. **b** Plasma glucagon levels (R&D ELISA kit) in 8-week male *C57BL/6* mice that were IP injected with galanin (0.32 mg/kg) with or without M35 (22 μg/kg) (*n* = 10). **c** Plasma glucagon levels (R&D ELISA kit) of STZ-treated mice that were IP injected with or without M35 (0.5 mg/kg/day) (*n* = 14 per group) as compared to control mice treated with PBS (*n* = 8). **d** Strategies of SD in mice using the water bath method. **e**–**i** Gut *GAL* mRNA (**e**), serum glucagon (**f**) (Sangon ELISA kit), hepatic *G6p* mRNA levels (**g**), body weight changes (**h**), and eWAT weights (**i**) of indicated 10-week mice. *C57BL/6* mice were IP injected with M35 (0.5 mg/kg/day) and GRA Ex-25 (GRA) (5 mg/kg/day) for 3 days and 1 day, respectively, before being transferred into a water bath platform for SD for 2 days (Con, *n* = 7; SD, *n* = 10; SD + M35, *n* = 11; SD + GRA, *n* = 10). **j**, **k** Body weight changes (**j**) and eWAT weights (**k**) of indicated mice with or without SD for 2 days (*Gcg*^*+/+*^, *n* = 8; *Gcg*^*+/+*^+ SD, *n* = 8; *Gcg*^*−/−*^+ SD, *n* = 8). **l**, **m** Hepatic PAS staining to indicate glycogen level (**l**) and H&E staining to indicate adipocyte mass (**m**) of indicated mice with SD and pharmaceutical intervention. Data were presented as means ± SEM. *P < 0.05.

Given the fact that Akh is functionally equivalent to mammalian glucagon mobilizing systemic energy storages^41^, we next ask whether a similar AstA/Akh axis also exists in SD mice to impair energy balance. Galanin receptors (GALR1/2/3), the mammalian homologs of AstA-R2, were shown to be activated by the gut hormone galanin^42,43^. To examine whether GALR signaling directly enhances glucagon release, we treated cultured mouse pancreatic α-cells (αTC1) with galanin and measured glucagon secretion. Interestingly, galanin increased glucagon release into the medium potently and mildly under low- and high-glucose conditions, respectively, without significantly affecting the expression of its receptors (Fig. 5a and Supplementary Fig. S7a). Incubation with M35, an established inhibitor of galanin receptors^44^, significantly suppressed galanin-induced glucagon secretion (Fig. 5a). We also examined the in vivo regulation of glucagon release by GALR signaling. We injected intraperitoneally (IP) galanin into *C57BL/6* mice that were fasted overnight and observed a significant increase in serum glucagon levels, which was further suppressed by M35 IP injection (Fig. 5b). Consistently, acute IP injection of galanin also caused hyperglycemia in wild-type (WT), but not *Gcg*^*−/−*^ mice (Supplementary Fig. S7b, c), indicating a glucagon-dependent role. Further, blockade of GALR signaling by M35 IP injection potently improved hyperglycemia, hyperglucagonemia, as well as glucagon-associated hepatic glycogen depletion, of streptozotocin (STZ)-treated mice with insulin deficiency^45^ (Fig. 5c and Supplementary Fig. S7d–g), excluding the participation of insulin. Taken together, our results demonstrate that, like AstA/Akh axis in *Drosophila*, mammalian galanin potentiates glucagon release in a conserved manner.

Finally, we assessed the impact of the galanin/glucagon axis on energy homeostasis in SD mice using a multi-platform of water bath^46^ (Fig. 5d). Interestingly, the intestinal expression of *Galanin* (*GAL*) gene, serum glucagon level, as well as hepatic target gene expression of glucagon, were significantly increased in the mice after 2-day SD (Fig. 5e–g). In line with the catabolic effects of glucagon on carbo-lipid storages, mice also exhibited weight loss, hepatic glycogen depletion, as well as lipid loss, under SD (Fig. 5h, i, l, m). Importantly, blockade of GALR signaling by M35 IP injection for 3 days prior to SD sufficiently decreased serum glucagon level and its associated metabolic activity, including hepatic target gene expression, hepatic glycogen depletion, lipid loss, and weight decline (Fig. 5f–i, l, m). To further verify the essential roles of glucagon, we deprived sleep of *Gcg*^*−/−*^ mice or blocked glucagon response using a small-molecule inhibitor, GRA Ex-25 (GRA). Consistent with M35 injection, either deficiency of glucagon or GRA IP injection sufficiently restored energy homeostasis, including lipid and glycogen contents and body weight, in the context of SD (Fig. 5h–m). Note that, glucagon deficiency in control mice with normal sleep hardly caused loss of body weight, adipose tissues, and hepatic glycogen (Supplementary Fig. S7h–k). Altogether, our results demonstrate that SD promotes galanin production, at least in the gut, to enhance glucagon release, leading to energy wasting.

### SD increases gut AstA production via ROS

**Fig. 6:**
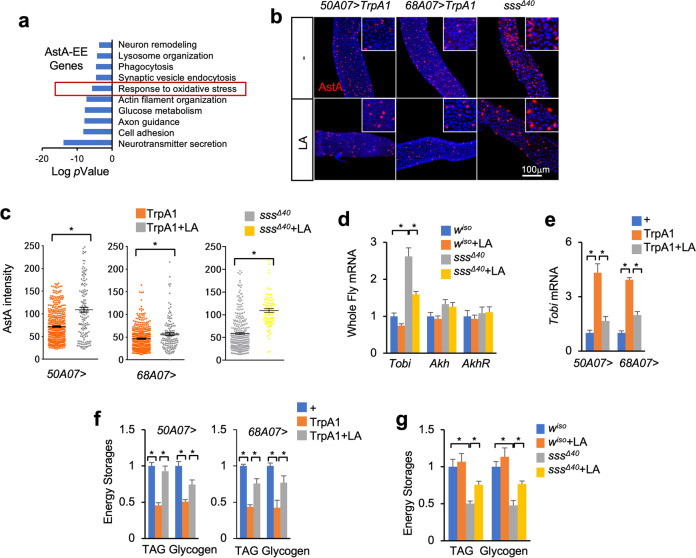
SD promotes gut AstA production via accumulated ROS. **a** Gene Ontology enrichment analysis of genes that are specifically expressed in the AstA-EE cluster. **b**, **c** Immunostaining of AstA (**b**) and quantification of intracellular AstA signal in each single cell (**c**, *n* > 100) in the posterior midgut of female flies of the indicated flies with SD for 6 days (TrpA1) or 6 days (mutant). **d**–**g** Whole-body gene expression (**d**, **e**) and TAG and glycogen storages (**f**, **g**) of indicated flies with 2 mM LA feeding in the food together with SD for 10 days (TrpA1) or 8 days (mutant) (*n* = 4, 5 flies/replicate). Data were presented as means ± SEM. *P < 0.05.

To explore the mechanisms of aberrant production in gut AstA by SD, we analyzed the gene expression pattern of AstA EEs from a published dataset of single-cell RNA sequencing (scRNA-seq)^16^. Among the 22 distinct cell clusters identified in the adult midgut, the AstA-EE cluster exhibits 1555 marker gene expression as compared to others (Supplementary Table S1). Gene Ontology enrichment analysis of these marker genes revealed a significant enrichment of genes involved in EE-associated cellular processes like “peptide secretion”, “vesicle endocytosis”, “cell adhesion”, and “glucose metabolism” (Fig. 6a). Interestingly, genes that are involved in “response to oxidative stress” are also significantly enriched (Fig. 6a). Since only severe (*50A07* > *TrpA1*, *68A07* > *TrpA1*, *sss*^*Δ40*^, mSD flies), but not moderate (*DAT*^*fmn*^) sleep loss triggers oxidative stress and ROS production in the adult fly midgut^8^ (Supplementary Figs. S1e, S8a–d), we wondered whether ROS triggers gut AstA production under SD. To address it, we fed different SD flies with the antioxidant lipoic acid (LA), which diminishes ROS accumulation^8^. Interestingly, as compared to controls, LA feeding significantly decreased gut ROS amount and increased intracellular AstA level in *50A07* > *TrpA1*, *68A07* > *TrpA1*, and *sss*^*Δ40*^ flies (Fig. 6b, c and Supplementary Fig. S8a–c), indicating decreased AstA release. LA feeding also decreased Akh release, systemic Akh response indicated by *tobi* expression, and loss of systemic glycogen and lipid in SD flies, but not in normal flies (Fig. 6d–g and Supplementary Fig. S8e).

### TrpA1 mediates ROS-induced gut AstA production

**Fig. 7:**
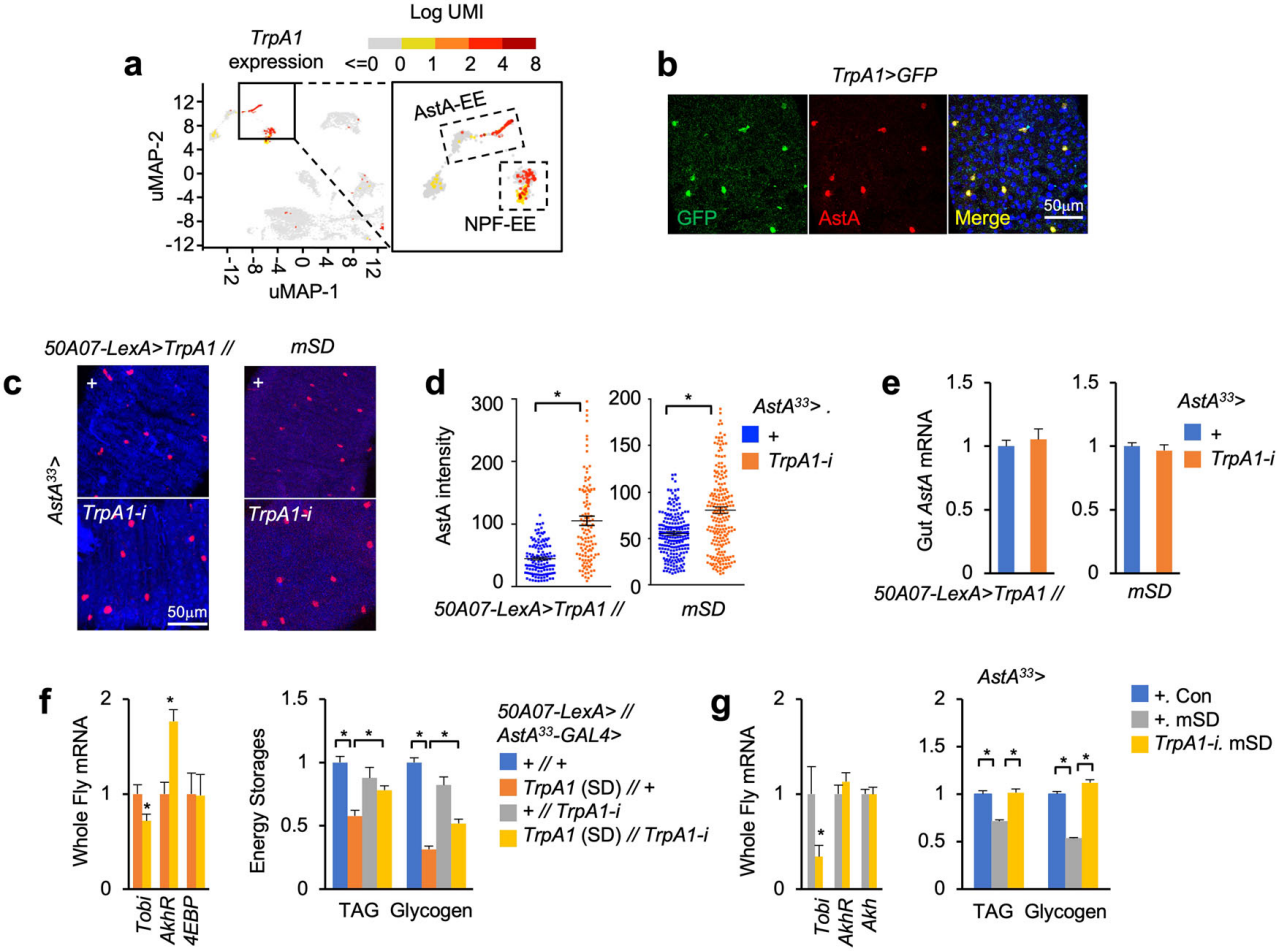
TrpA1 mediates gut AstA release in SD flies. **a** UMAP of *TrpA1* expression in different cell clusters of adult midguts. **b** Immunostaining of AstA (red) and TrpA1>GFP (green) expression in the posterior region of the adult midgut. **c**–**g** Immunostaining of AstA (**c**) and quantification of intracellular AstA signal in each single cell (**d**, *n* > 100) in the posterior midgut, gut *AstA* mRNA (**e**, *n* = 3, 15 flies/replicate), whole-body gene expression (**f**, **g**, left, *n* = 4, 5 flies/replicate), and TAG and glycogen storages (**f**, **g**, right, *n* = 4, 5 flies/replicate) of the indicated genotypes after SD for 10 days (TrpA1) or 8 days (mSD). Data were presented as means ± SEM. *P < 0.05.

Through scRNA-seq analysis, we further identified *TrpA1*, a major ROS sensor controlling gut hormone release^47^, as a top gene that is specifically expressed in AstA EEs (Fig. 7a). We examined GFP expression driven by a *TrpA1-GAL4* and observed that it is co-localized with AstA expression in the posterior midgut (Fig. 7b). Using knock-in *T2A-GAL4* lines that report the expression of individual *TrpA1* isoforms^48^, we also revealed the expression of *TrpA1-C* and *-D* that respond to ROS in AstA EEs (Supplementary Fig. S8f)^49^. To examine whether TrpA1 regulates AstA production in response to ROS, we knocked down *TrpA1* specifically in the AstA EEs of both thermogenetic *50A07* > *TrpA1* and mSD flies. We strikingly found that AstA release and Akh-induced *tobi* induction were decreased and that energy-wasting phenotypes, including TAG and glycogen depletion, were remarkably improved (Fig. 7c–g). Note that, *TrpA1* knockdown in AstA EEs of control flies with normal sleep failed to elevate systemic TAG and glycogen storages (Fig. 7f). Taken together, these results indicated that ROS promotes gut AstA production via TrpA1 in SD flies.

## Discussion

**Fig. 8:**
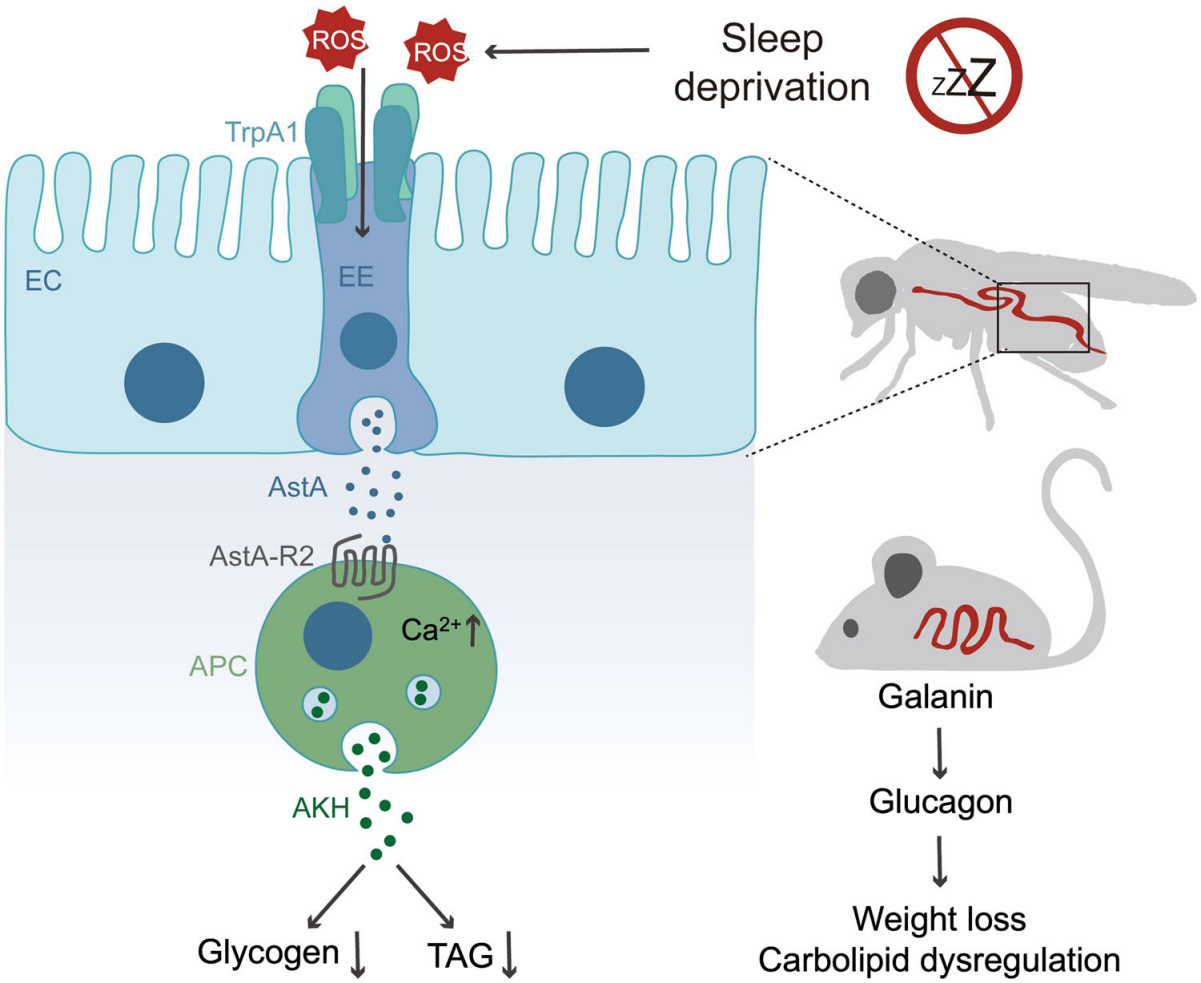
The schematic model of SD-induced energy loss in both flies and mice. Fly AstA EEs express TrpA1 to sense SD-induced ROS in the gut lumen and enhance AstA production to remotely target Akh-producing cells via AstA-R2, leading to increased Akh secretion and systemic energy mobilization. Similar AstA/galanin-triggered Akh/glucagon secretion and energy loss is observed in sleep-deprived mice.

In this study, we uncover that SD enhances the production of gut AstA to cause energy wasting. The molecular mechanisms include that, at least, gut AstA remotely targets APCs to promote Akh release, leading to systemic loss of lipid and glycogen. We also show similar regulation in SD mammals whereby galanin signaling enhances glucagon secretion to cause energy wasting. Finally, we demonstrate that gut AstA EEs sense the intestinal ROS through TrpA1 to increase AstA production (Fig. 8).

Sleep loss has been highly associated with metabolic dysregulation^1^; however, the metabolic output in different SD flies remains controversial in *Drosophila*. Specifically, a decline of whole-body TAG storages has only been observed in severe sleep-loss flies (*50A07* > *TrpA1*, *68A07* > *TrpA1*, *sss*^*Δ40*^, and mSD), but not in moderate sleep-loss (*DAT*^*fmn*^) flies. A recent study reported extensive ROS accumulation only in the guts of flies under severe, but not moderate, sleep loss^8^. In this study, we consistently found that the diminishment of gut ROS by feeding of LA, a ROS scavenger, sufficiently restores energy storages in severe sleep-loss flies, indicating the important roles of gut ROS in systemic energy maintenance. We further elucidated the mechanisms that gut ROS promotes the production of a gut-peptide hormone AstA to affect systemic carbo-lipid metabolism in an endocrinal manner. In support of our notion, intestinal AstA production was found to be increased only in severe sleep-loss flies. Thus, our results demonstrate ROS/AstA axis in the gut as the key pathogenic factor mediating metabolic dysregulation in various SD flies.

Genes encoding gut-peptide hormones are also expressed in the brain of *Drosophila*^18^. It is essential to use specific drivers to investigate the functions and regulations of EE-derived gut hormones such as Tk and NPF, distinguishing from the ones produced in the brain. Many studies managed to use *pros-GAL4* that targets all EEs in the gut to knock down the expression of peptide hormones^21,34^. However, it is difficult to perform loss-of-function in certain subtype EEs for investigating physiological regulations such as nutrient or stress signals of gut-peptide production. We have previously generated a specific *Tk-g-GAL4* that predominantly expresses in the gut Tk EEs, and that allows us and other groups to reveal the metabolic functions and regulations of Tk in the gut^19,20,22,50,51^. In this study, we further generated a specific *AstA*^*33*^*-GAL4* that predominantly targets AstA EEs in the gut. We characterized the critical roles of gut AstA as mobilizing systemic lipid and carbohydrate storages under SD integrating *AstA*^*33*^*-* and *pros-GAL4*, even though the participation of AstA derived by certain neurons was not completely excluded. Moreover, we used this *AstA*^*33*^*-GAL4* to uncover TrpA1 as an important regulator sensing intracellular ROS to promote AstA production under SD. Since multiple functional receptors and stress sensors are found to be expressed in the gut EEs to orchestrate the hormone production in the context of physiological and environmental cues^11^, we believe our *AstA*^*33*^*-Gal4* will offer opportunities to exploit diverse regulation of AstA release under SD and beyond.

Even though AstA-R2 was found to be expressed in APCs and to regulate Akh release^25^, the origin(s) of functional AstA was not well characterized. Here we confirmed the interaction between gut AstA and Akh in APCs genetically, as *AstA-R2* knockdown in APCs plus *AstA* knockdown in EEs failed to further impair energy balance as compared to the knockdown of each of them individually. Similar results were obtained by performing *Akh* deficiency plus *AstA* knockdown in EEs. On the other hand, it has been reported that the production of Akh, which functions as a central metabolic hormone, is both positively and negatively modulated by multiple gut-peptide hormones like NPF and Burs^21,34,51^, highlighting a sophisticated Yin-Yang maintenance of Akh release and systemic energy homeostasis. It will be important to investigate whether other gut-peptide hormones also participate in SD-induced energy loss.

*TrpA1* encodes an ion channel sensing extracellular stresses, including ROS, to activate Ca^2+^ signaling and regulate diverse biological processes in *Drosophila*^47,49^. In this study, we found that both AstA release and AstA-EE numbers were significantly increased by gut ROS in SD flies. Note that, *TrpA1* knockdown in AstA EEs using *AstA*^*33*^*-GAL4* diminished the increase only in AstA release but not in AstA-EE numbers of SD flies, demonstrating the essential roles of TrpA1 in AstA EEs to sense ROS and promote AstA release. It is in line with previous studies of TrpA1 expression in EEs and TrpA1 regulation of DH31 release^47^. Previous studies have shown that TrpA1-associated Ca^2+^ signaling in ISCs is required for both self-proliferation and differentiation into EEs^49,52^; therefore, it is possible that ROS impacts ISCs via TrpA1 to increase AstA-EE number. We performed *TrpA1* knockdown in ISCs of *50A07* > *TrpA1* flies and, however, failed to observe any changes of AstA-EE numbers, AstA production, or carbo-lipid metabolism (data not shown), excluding TrpA1 roles in ISC differentiation into AstA EEs in SD flies.

Similar to AstA/Akh axis in *Drosophila*, we also confirmed that galanin-mediated glucagon release is essential for SD-induced energy loss in mammals, indicating an evolutionarily conserved phenomenon. Previous studies have suggested that galanin directly targets β-cells and inhibits the secretion of insulin that potently suppresses glucagon release^53^, generating a plausible hypothesis that galanin indirectly enhances glucagon secretion via diminishing insulin-leashed glucagon release. Here, we provide evidence that galanin directly impacts glucagon secretion. First, galanin promotes glucagon secretion in cultured α-cells and triggers glucagon-dependent hyperglycemia in mice. Second, blocking galanin signaling sufficiently impairs glucagon secretion and glycemic elevation under conditions with impaired insulin response, including chronic fasting and STZ-induced β-cell loss. In support of our conclusion, elevations of blood galanin, glucagon, and glucose levels were observed in both type 1 and 2 diabetic patients with impaired insulin response^54–56^. Nevertheless, mammalian Kiss 1 receptor (KISS1R) also shares homology (albeit lower than GALRs) with AstA-R2 as indicated by DIOPT Ortholog Finder^57^; it would be interesting to study the roles of KISS1R signaling in SD-induced energy wasting as well. Altogether, our results reveal that gut galanin modulates glucagon release to augment energy wasting in SD mice and provide novel pharmaceutical opportunities for the treatment of metabolic disorders in SD patients.

## Materials and methods

### Drosophila Strains

*UAS-Rpr* (#5823), *Akh-GAL4* (#25684), *UAS-srcGFP* (#5432), *UAS-CaLexA-GFP* (#66542), *UAS-TrpA1* (#26264), *UAS-AstA-RNAi-JF* (JF01905, #25866), *TrpA1-GAL4* (#36922), *UAS-AR2-RNAi-JF* (JF01955, #25935), *elav-GAL4/CyO,GFP* (#8765), *AstA-R2-GAL4* (#76727), *11H05-GAL4* (#45016), *50A07-LexA* (#61586), and *68A07-LexA* (#53538) were obtained from Bloomington Drosophila Stock Center (BDSC). *UAS-AstA-R2-i-NIG* (NIG10001R-1) and *UAS-AstA-i-v* (v103215) were obtained from Fly Stocks of the National Institute of Genetics (NIG, Japan) and Vienna Drosophila Resource Center (VDRC), respectively. *Akh*^*SAP*^ and *Akh*^*A*^ were kind gifts from Dr. Ronald Kühnlein (University of Graz);^39^ *sss*^*Δ40*^, *DAT*^*fmn*^, and matched control flies *w*^*iso31*^ from Dr. Chang Liu (Shenzhen Institutes of Advanced Technology, China); *LexAop-TrpA1* from Dr. Yufeng Pan (Southeast University, China); *KI-T2A-GAL4* lines for different *TrpA1* isoforms from Drs. Yang Xiang (UMass Chan Medical School) and Pengyu Gu (Southeast University, China)^48^. Negative controls, *w*^*1118*^ and *yv;attp2*, exhibited similar phenotypes. More information on flies used in this study was included in Supplementary Table S2.

The promoter region of the *AstA* gene was cloned by classical PCR using primers, 5′-GCGCAATTGGGGAAAAATCTCCGAAAACC-3′ and 5′-GCCGGATCCAGAGGTTCCGCGGACTAAAT-3′, to generate different *AstA-GAL4* flies. We crossed these *AstA-GAL4* lines to *UAS-srcGFP* to examine the expression patterns and used *AstA*^*33*^*-GAL4* that targets all AstA EEs but very limited AstA neurons in this study.

Flies were maintained on food, which contained 30 g yeast, 50 g corn flour, and 50 g Maltose in 1 L distilled water, in 65% humidity and 25 °C incubators in a 12-h light 12-h dark (12:12 LD) cycle. For LA treatment, virgin flies were transferred onto food containing 2 mM LA (A506197, Sangon Biotech) every 2 days. The genotypes of flies used in this study were included in Supplementary Table S3.

### SD and sleep measurement in flies

Thermogenetic SD was performed using the Gal4/UAS and LexA/LexAop systems. Flies were raised in 12:12 LD cycles at 18 °C until 6–7-day post eclosion. The temperature was raised to a constant 29 °C, which triggered SD. Deprivation was done in 12:12 LD cycles.

mSD was performed at 25 °C using a multi-tubes vortexer. Flies were raised at 25 °C (12:12 LD) until day 4–5 post eclosion. After a day of baseline sleep recording, the multi-tube vortexer delivered 10-s vibrations at intervals centered around 50 s. The intensity of the vortexer was set to 4 (~1500 rpm).

5-day old female flies were transferred to separated glass tubes containing 5% sucrose agar food, then the tubes were sealed with a ventilated rubber plug. The tubes were inserted into *Drosophila* activity monitors to measure activities under a 12:12 LD at 25 °C or 29 °C.

### Mice

Male *Gcg*^*−/−*^ (T014382, Gempharmatech) and *C57BL/6* mice were maintained at room temperature (21 ± 2 °C), the humidity of 50% ± 15%, and a 12-h cycle of light and dark in the animal facility of Medical Research Institute, Wuhan University and fed either a normal chow diet ad libitum with free access to water. All animal experiments were performed under animal ethical regulations and the study protocol was approved by the Institutional Animal Care and Use Committee of Wuhan University.

We IP injected 0.32 mg/kg galanin (T510180, Sangon Biotech) with or without 22 μg/kg M35 (GWTLNSAGYLLGPPPGFSPFR-NH2, purity: 98%, Cusabio) dissolved in PBS into 8–10-week-old *C57BL/6* and *Gcg*^*−/−*^ male mice at the overnight-fasting state. To disrupt the integrity of pancreatic β-cells, we IP injected 40 mg/kg/day STZ (T1507, TargetMol, USA) dissolved in 0.1 M citrate buffer (pH 4.5) into *C57BL/6* mice under a fasting state for five consecutive days. Mice with blood glucose higher than 16.7 mM or 300 mg/dL were characterized as diabetic subjects for subsequent IP injection of 0.5 mg/kg/day M35 dissolved in PBS. As for SD, mice were transferred to a multiple-platform water bath with free access to water and food for 48 h under controlled conditions of temperature (22–24 °C), humidity (55%–60%), and a 12:12 LD cycle. Multiple small platforms (2.5 cm) were placed in a tank (40 × 30 cm) filled with water to within 1–4 cm of the upper surface of the platforms and spaced 4 cm. Mice were IP injected with 0.5 mg/kg/day M35 to block galanin response for 3 days or 5 mg/kg/day GRA-Ex25 (T3422 TargetMol, USA) to block glucagon resonse for 1 day prior to SD. Orbital blood samples were collected under anesthesia to examine serum glucagon levels using an ELISA kit (DGCG0, R&D Systems; D721189, Sangon Biotech) according to the standard protocol. Each group contained at least eight mice.

### Generation of anti-Akh antibodies

Polyclonal antibodies against *Drosophila* Akh were prepared by Dai-An Biotech in Wuhan, China. The Akh peptides (CQLTFSPDWa), which were conjugated to keyhole limpet hemocyanin (KLH) through the added N-terminal cysteine, were immunized in rabbits four times. The antisera were purified by an affinity column coupled with Akh peptides. The polyclonal Akh antibodies have been diluted and sourced to a commercialized company as an OEM product (A22867, Abclonal).

### Immunostaining, lipid staining, and microscopy

Gut, brain, and fat-body tissues from adult flies were dissected in PBS and fixed for 15 min in 4% formaldehyde/PBS. After fixation, the samples were washed with 0.2% Triton/PBS and incubated in primary antibody overnight at 4 °C. After incubation of second antibody (1:1000, Alexa Fluor, Thermo Fisher) plus DAPI (1:1000, Thermo Fisher Scientific) at room temperature for 1 h, tissues were washed and mounted in Antifade Mounting Medium (P0128M, Beyotime). Primary antibodies used in this study included anti-AstA (1:2000)^58^, anti-Tk (1:5000)^19^, anti-Prosper (1:100, MR1A, Developmental Studies Hybridoma Bank), anti-Akh (1:1000, A22867, ABclonal). Bodipy 493/503 (1 mg/mL, D3922, Thermo Fisher Scientific) were used for staining of neutral lipids.

For DHE staining, adult *Drosophila* guts were dissected with PBS containing 2 μM DHE (S0063, Beyotime) medium at room temperature in darkness for 15 min. Before mounting, samples were briefly washed with PBS three times and fixed with 4% paraformaldehyde at room temperature for 20 min.

Dissected mouse liver samples were fixed in 10% neutral buffered formalin for 48 h and performed Periodic acid Schiff (PAS) staining to detect glycogen by Wuhan Servicebio Biotechnology according to a standard protocol. Microscopy was performed on an inverted Zeiss LSM880 laser scanning confocal microscope. For quantification of AstA, Akh, and CaLexA staining intensities, stacks were *Z*-projected, and the signals of AstA in each midgut cell and Akh or CaLexA (GFP) in the whole CC were quantified using Integrated Density in ImageJ. The background was subtracted to give the net signal.

### Cell culture and glucagon secretion

αTC1 cells were cultured in DMEM (PM150211, ProCell, China) supplemented with 1 g/L glucose, 10% FBS and antibiotics. After incubation with 100 nM galanin with or without 1 nM M35 for 4 h, αTC1 cells were washed and cultured with DMEM containing different doses of glucose for 4 h. The supernatants were collected and centrifuged at 5000× *g* for 5 min at 4 °C to remove cells and debris. Glucagon contents in the supernatants were detected using an ELISA kit (DGCG0, R&D Systems).

### qPCR

Fifteen brains, 15 midguts, or five adult flies from each genotype and αTC1 cells were lysed to extract total RNA using Trizol (Invitrogen). cDNA synthesis was performed using HiScript II reverse transcriptase supermix (R223-01, Vazyme). Quantitative real-time PCR was performed using ChamQ SYBR master mix (Q311-02, Vazyme) on a CFX384 Real-Time System/C1000 Thermal Cycler (BioRad). Fly and mouse gene expressions were normalized to *RpL32* and *β-actin*, respectively. qPCR primers used in this study are listed in Supplementary Table S4.

### TG and glycogen measurement

Five flies that were fed or starved from each group were homogenized with 0.5 mL PBS containing 0.2% Triton X-100 and heated at 70 °C for 5 min. The supernatant was collected after centrifugation at 12,000 rpm for 10 min at 4 °C. Ten microliters of supernatant was used for protein quantification using Bradford Reagent (E111-01, Vazyme). Whole-body glycogen levels were measured from 10 μL of supernatant treated with or without 0.4 μL Amyloglucosidase (2 mg/mL, A7420, Sigma) to degrade glycogen into glucose at 37 °C for 30 min using glucose assay reagent (K-GLUC, Megazyme) following the manufacturer’s protocol. We subtracted the amount of free glucose from the measurement and then normalized the subtracted values to protein levels in the supernatant. To measure whole-body triglycerides, we processed 10 μL of supernatant using a Serum Triglyceride Determination kit (TR0100, Sigma). We subtracted the amount of free glycerol in the supernatant from the measurement and then normalized the subtracted values to protein levels in the supernatant.

### Dot-blot analysis of hemolymph Akh

Forty fed or 60 starved flies were decapitated and centrifuged at 1000× *g* for 15 min at 4 °C in the 0.6 mL Eppendorf tube embedding in a 1.5 mL Eppendorf tube. About 0.1 μL hemolymph was diluted into 10 μL PBS and loaded onto 0.2 μM nitrocellulose membrane (GE Healthcare) and air-dried for 20 min. The membrane was subsequently boiled in PBS for 3 min, fixed with 4% PFA for 20 min, and recovered in PBS for 5 min at room temperature. After Ponceau Red staining as loading control, the membrane was blocked with 5% BSA in PBS for 1 h and incubated with purified anti-Akh antibodies (1:1000, Abclonal, A22867) in 5% BSA at 4 °C overnight, followed by incubation with horseradish peroxidase (HRP)-conjugated secondary antibodies in 5% BSA in PBS for 1 h at room temperature. The membrane was developed using X-ray film in the darkroom.

### Mortality under starvation

Adult flies of each genotype were aged for 5–6 days at 25 °C then transferred into 1% agar food (just agar and H_2_O). Dead flies were counted every 12 h.

### Statistical analysis

Data were representative of observations made in at least two independent experiments. Data were presented as the means ± SEM. Unpaired Student’s *t*-test and one-way ANOVA followed by post hoc test were performed to assess differences. A *P* value of < 0.05 was considered statistically significant.

## Supplementary information

Supplementary Information

Supplementary Table S1
