# Supplementary material for: Gut AstA mediates sleep deprivation-induced energy wasting in Drosophila
Source: Cell Discov. 2023 May 23;9:49. doi: 10.1038/s41421-023-00541-3 (PMC10206085; doi:10.1038/s41421-023-00541-3)
Supplement: Supplementary file 1 — Supplementary Information [file 41421_2023_541_MOESM1_ESM.pdf]

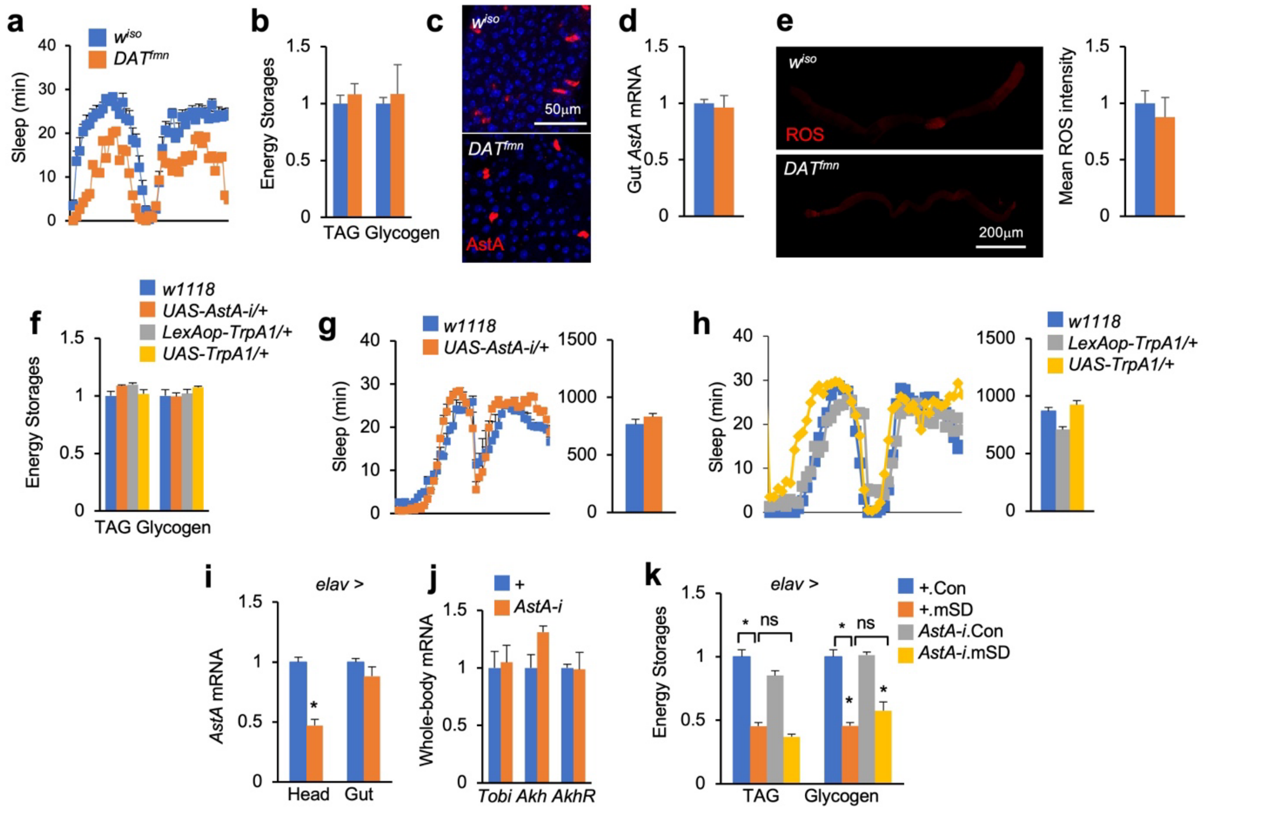

Supplementary Figure 1. Moderate sleep loss or brain AstA does not affect energy wasting (related to Fig. 1). (**a-e**) Sleep in every 30 min per day (**a,** *n* = 29), TAG and glycogen storages (**b,** *n* = 4, 5 flies/replicate), gut AstA immunostaining (**c,** left, red), gut *AstA* mRNA level (**d,** *n* = 3, 15 midguts/replicate), as well as ROS staining in the gut (**e,** left, red) and quantification (**e,** right, *n* = 5), in the control and DAT^{fmn} flies. (**f-h**) TAG and glycogen storages (**f,** *n* = 4, 5 flies/replicate), sleep in every 30 min and total sleep per day (**g-h,** left, *n* = 16) of indicated 5-day old flies. (**i-k**) AstA mRNA levels in brain (**i,** *n* = 4, 5 flies/replicate) and gut (**i,** *n* = 4, 10 flies/replicate), whole-body mRNA levels (**j,** *n* = 4, 5 flies/replicate), and TAG and glycogen storages (**k,** *n* = 4, 5 flies/replicate) of indicated 5-day old flies with mSD for 8 days. Data are presented as mean ± SEM. **P* < 0.05.

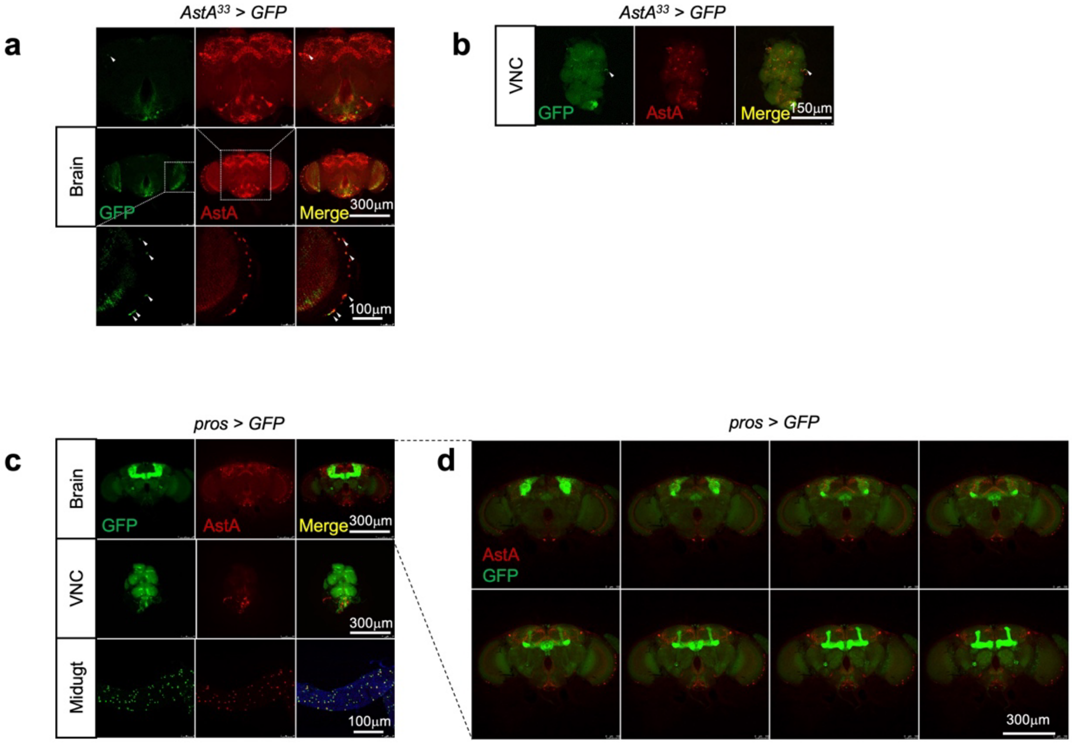

Supplementary Figure 2. Expression patterns of *AstA*^{33}*-* and *pros-GAL4* in the brain, VNC, and midgut (related to Fig. 2). (**a-b**) Immunostaining of endogenous AstA (red) and AstA^{33}>GFP expression (green) in the regions of protocerebrum (**a,** top), fan-shaped body and mushroom body (**a,** middle), and optic lobe (**a,** bottom) in the brain and VNC (**b**) of 6-day old female. Arrow heads indicate the colocalization of AstA^{33}>GFP and endogenous AstA signals. (**c-d**) Z-stack confocal images of immunostaining of endogenous AstA (red) and GFP expression (green) driven by *pros-GAL4* in the brain (**c,** top; **d**, single-projection images of brain), VNC (**c,** middle), and posterior midgut (**c,** bottom) of 6-day old female.

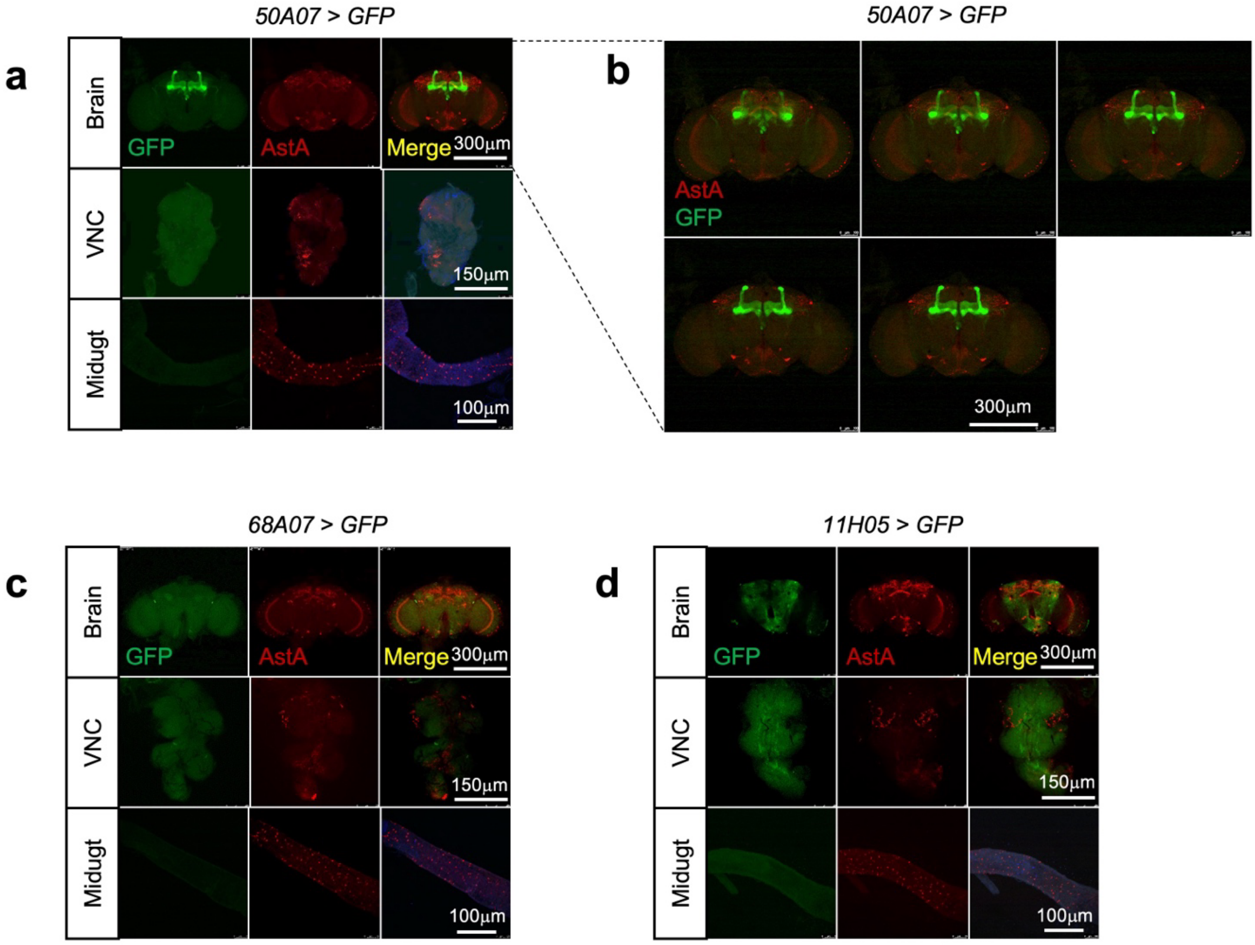

Supplementary Figure 3. Expression patterns of different drivers in the brain, VNC, and midgut (related to Figs. 1 and 2). Confocal images of immunostaining of endogenous AstA (red) and GFP expression (green) driven by *50A07-LexA* (**a,** different tissues; **b,** single-projection images of brain), *68A07-LexA* (**c**), and *11H05-LexA* (**d**) in the brain (top), VNC (middle), and posterior midgut (bottom) of 6-day old female.

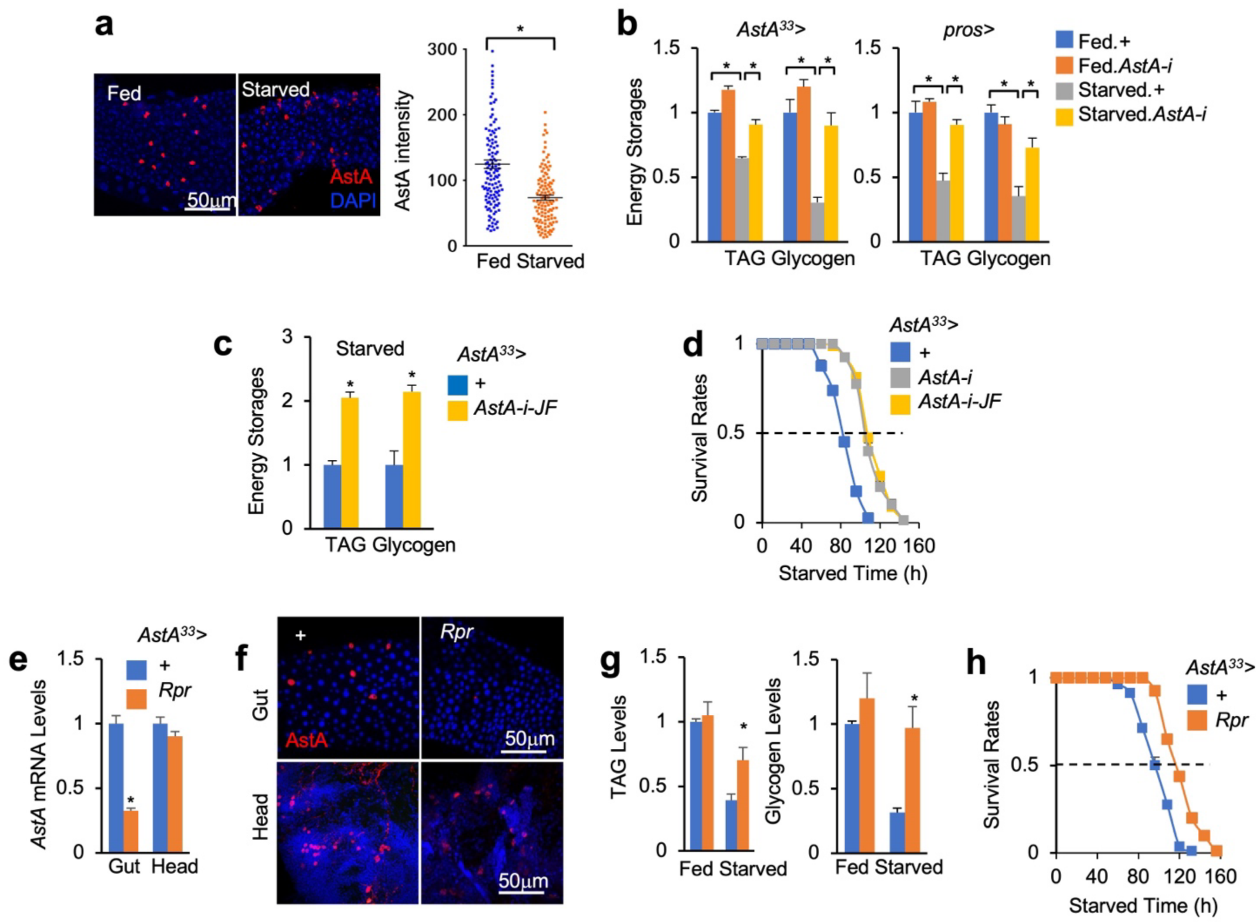

Supplementary Figure 4. Gut AstA deficiency increases TAG and glycogen storages (related to Fig. 3). (**a**) Immunostaining of AstA (left) and quantification of intracellular AstA signal/cell (right, n > 100) in the gut of 7-day old females that were fed or starved for 36 h. (**b-c**) TAG and glycogen storages (n = 4, 5 flies/replicate) of 7-day old females that were fed or starved for 36 h with indicated genotypes. (**d**) Survival rates under starvation of indicated flies (n = 4, 20 flies/replicate). (**e-f**) *AstA* mRNA (**e,** n = 3, 15 midguts/replicate) and protein (**f**) levels in the midguts and heads of indicated female flies at day 7 (n = 3, 5 flies/replicate). (**g**) TAG and glycogen storages (n = 4, 5 flies/replicate) of 7-day old females that were fed or starved for 48 h with indicated genotypes. (**h**) Survival rates under starvation of indicated flies (n = 4, 20 flies/replicate). Data are presented as mean ± SEM. *P < 0.05.

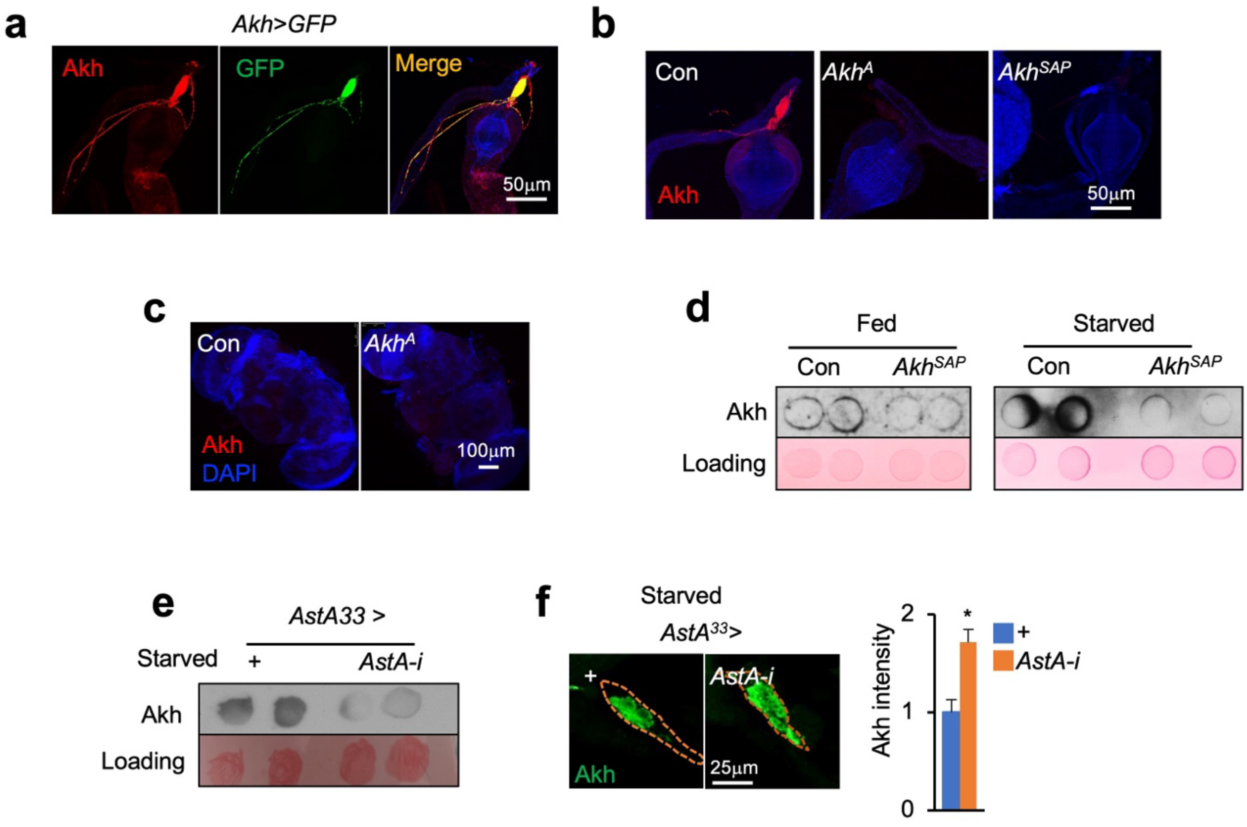

Supplementary Figure 5. Gut AstA promotes Akh release (related to Fig. 4). (**a**) Immunostaining of Akh (red) and Akh>GFP (green) expression in the APCs of 8-day old female flies. (**b-c**) Immunostaining of Akh (red) in the APCs (**b**) and brain (**c**) of indicated 8-day old female flies. (**d**) Dot-blot assays indicating circulating Akh levels in the hemolymph of indicated 7-8-day old female flies that were fed or starved for 48 h (30 flies/pool). (**d-e**) Dot-blot assays indicating circulating Akh levels in the hemolymph (**f**) and confocal images of intracellular Akh in APCs (**f**, left, images; right, quantification of Akh signal/fly, n = 4) of indicated 7-8-day old female flies that were starved for 48 h (30 flies/pool). Data are presented as mean ± SEM. *$P < 0.05$.

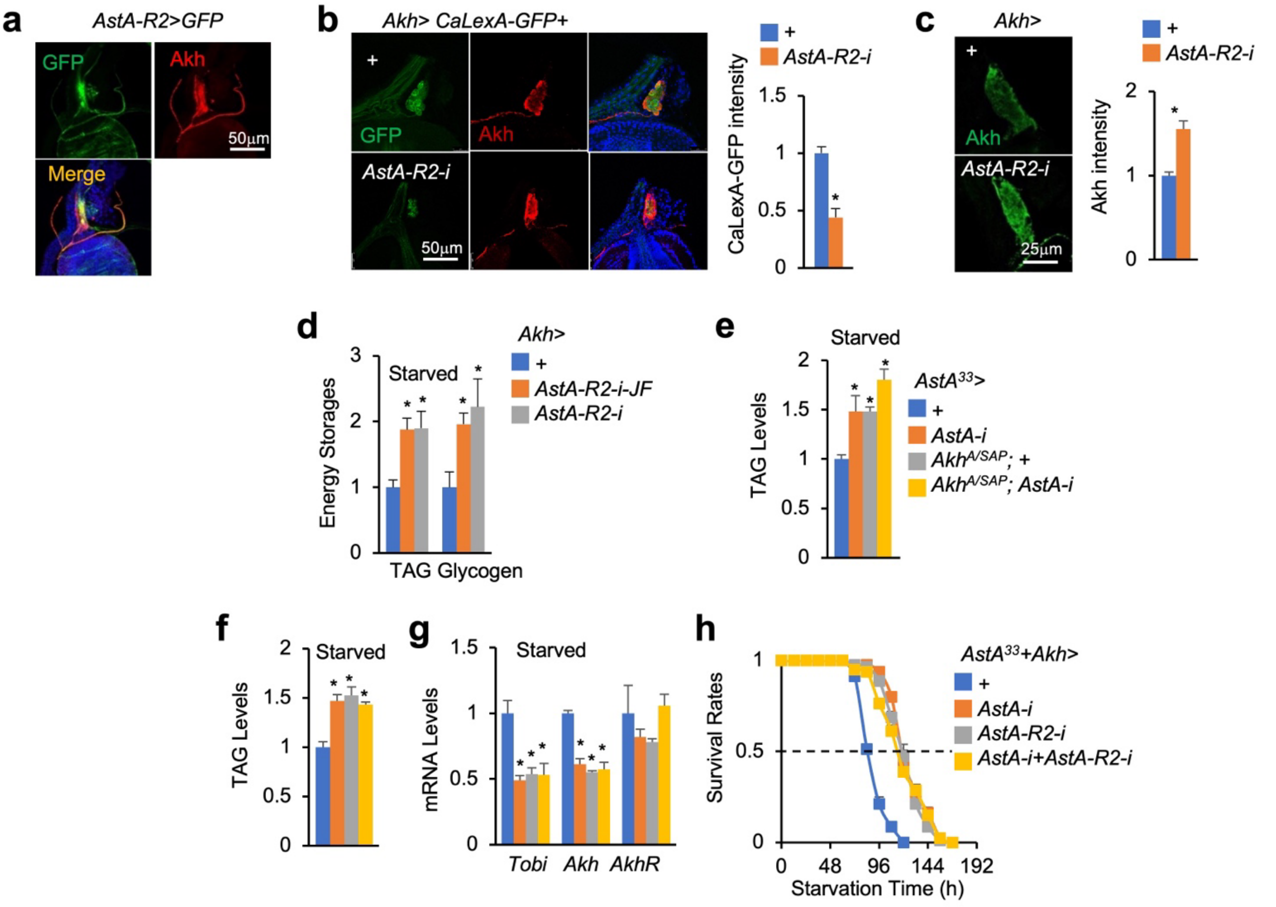

Supplementary Figure 6. Gut AstA promotes Akh release via AstA-R2 (related to Fig. 4). (**a**) Immunostaining of Akh (red) and AstA-R2>GFP (green) expression in the APCs of 8-day old female flies. (**b-d**) *AstA-R2* knockdown in APCs regulates intracellular Ca^{2+} level as indicated by CaLexA-GFP (**b**, left, green; right, quantification, *n* = 6), intracellular Akh levels (**c**, left, green; right, quantification, *n* = 10), and TAG and glycogen storages (**d**, *n* = 4, 5 flies/replicate) of indicated flies under starvation for 48 h. (**e**) TAG storages of indicated flies under starvation for 48 h (*n* = 4, 5 flies/replicate). (**f-h**) TAG storages (**f**, *n* = 4, 5 flies/replicate), gene expression in the whole body (**g**, *n* = 4, 5 flies/replicate), as well as survival rates (**h**, *n* = 4, 20 flies/replicate), of indicated 8-day old female flies under fed or starved condition. Data are presented as mean ± SEM. *$P < 0.05$.

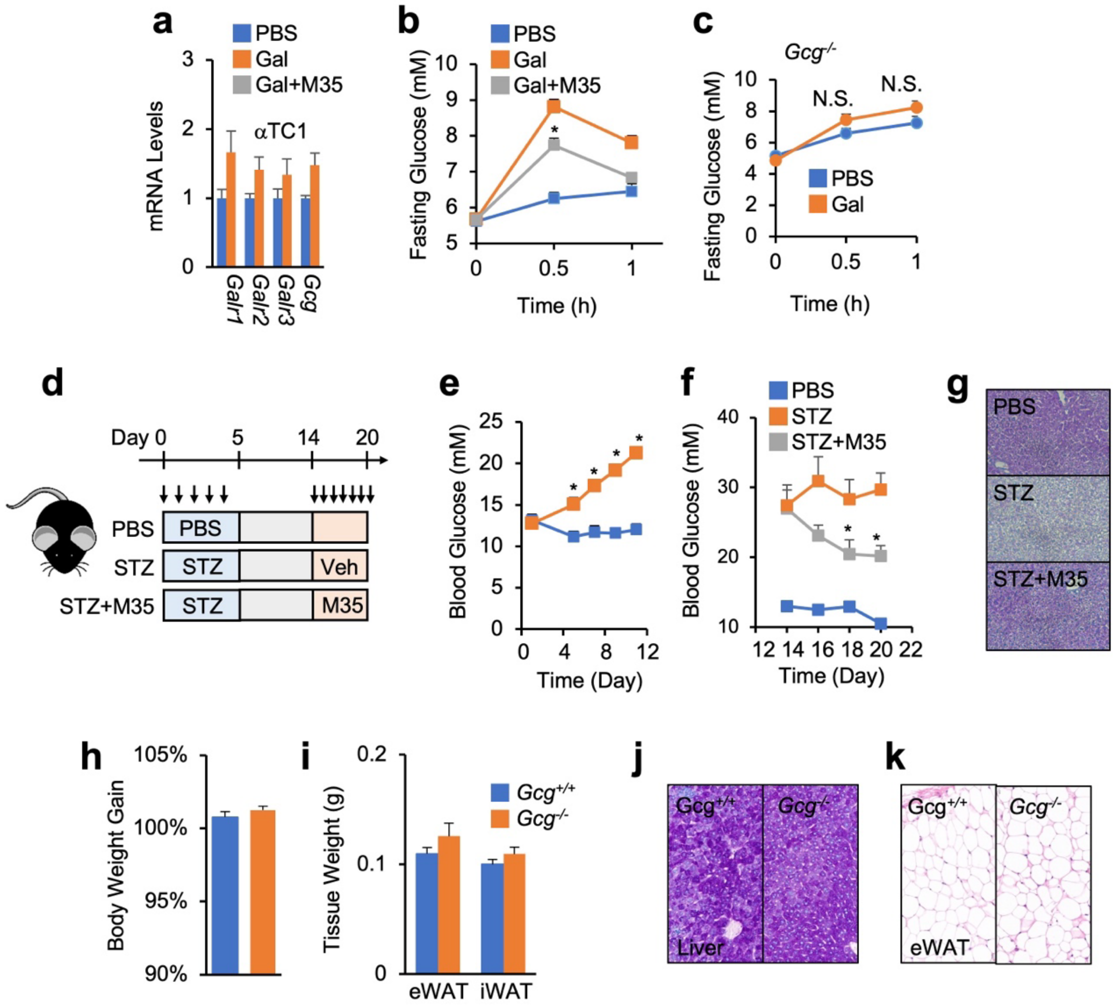

Supplementary Figure 7. GALR signaling regulates carbo-lipid metabolism through glucagon functions (related to Fig. 5). (**a**) mRNA levels in αTC1 cells that were treated with 100 nM galanin (Gal) for 4 h. (**b-c**) Fasting blood glucose of 8-week-old male *C57BL/6* (**b**, *n* = 10) or *Gcg*$^{-/-}$ mice (**c**, *n* = 7) that were IP injected with galanin (Gal) (0.32 mg/kg) with or without M35 (2.2 μg/kg). (**d**) The strategy for generation of β-cell-impaired diabetic mice. (**e-g**) Basal blood glucose levels (**e-f**) and liver glycogen contents indicated by PAS staining (**g**) of STZ-treated mice that were IP injected with (*n* = 14) or without M35 (0.5 mg/kg/day) (*n* = 14), healthy 7-week mice treated with PBS (*n* = 8). (**h-i**) Body weight changes (**h**), WAT weights (**i**, *Gcg*$^{+/+}$, *n* = 7; *Gcg*$^{-/-}$, *n* = 7), hepatic PAS staining to indicate glycogen level (**j**) and H&E staining to indicate adipocyte mass (**k**) of indicated 7-week mice without sleep deprivation for 2 days. Data are presented as mean ± SEM. *P < 0.05.

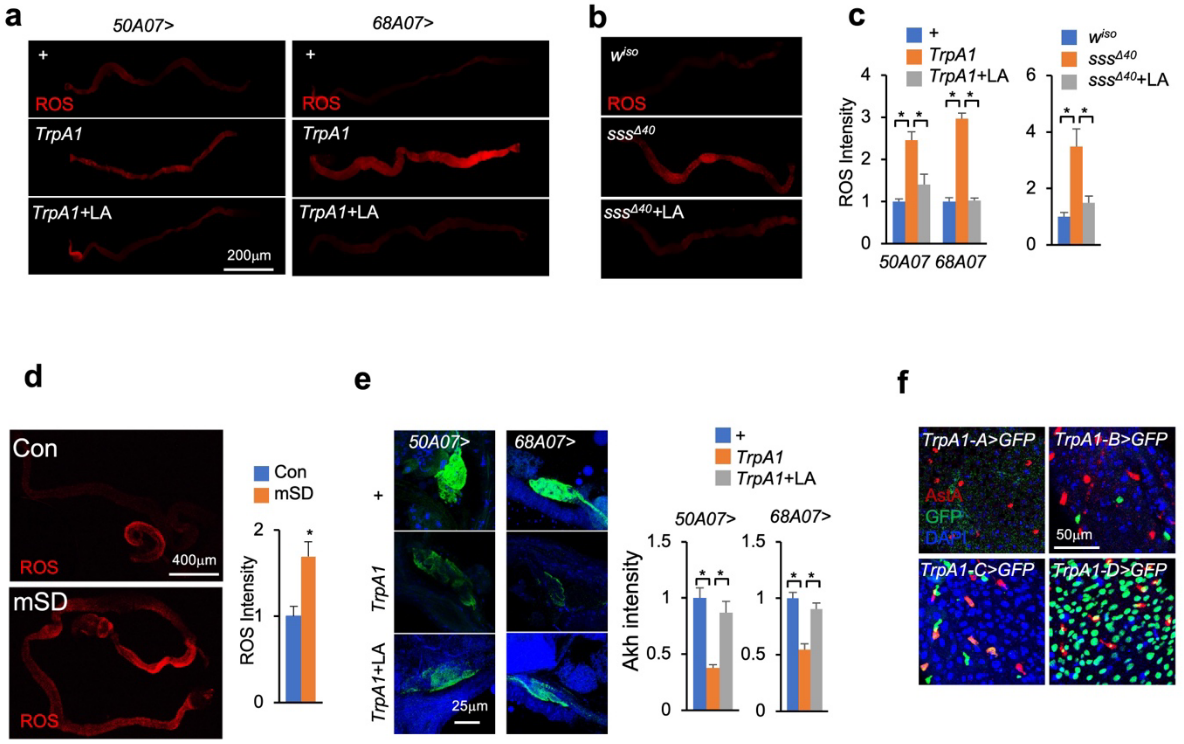

Supplementary Figure 8. ROS accumulation and Akh production in sleep-loss flies (related to Figs. 6 and 7). (**a-b**) Intestinal ROS staining (red) in the whole midgut of the indicated flies fed with or without 2 mM LA together with sleep deprivation for 10 days (**a,** TrpA1) or 8 days (**b,** mutant). (**c**) Qualification of ROS intensity in the indicated genotypes (n = 5). (**d**) Intestinal ROS staining in the whole midgut of mSD flies (left, red; right, quantification, Con, n = 6; mSD, n = 10). (**e**) Intracellular Akh level (left, green) and quantification (right, n = 6) of the indicated genotypes fed with or without 2 mM LA. (**f**) Immunostaining of endogenous AstA (red) and GFP (green) expression driven by *KI-T2A-GAL4* lines of different isoforms of *TrpA1* in the posterior region of adult midgut. Data are presented as mean ± SEM. *P < 0.05.

Supplementary Table S2. Key resources.

**REAGENT or RESOURCE**	**Source**	**IDENTIFIER**
**Fly stocks**		
68A07-LexA	Bloomington Stock Center	BDSC_53538
50A07-LexA	Bloomington Stock Center	BDSC_61586
w^{iso31}	Gift from Dr. Chang Liu	Previous study [1]
$sss^{\triangle 40}$	Gift from Dr. Chang Liu	Previous study [1]
DAT^{fmn}	Gift from Dr. Chang Liu	Previous study [2]
LexAop-TrpA1 (VK5)	Gift from Dr. Yufeng Pan	Previous study [3]
$AstA^{33}$*-GAL4*	This study	N/A
pros-GAL4	Our previous study	Previous study [4]
elav-GAL4/CyO,GFP	Bloomington Stock Center	BDSC_8765
11H05-GAL4	Bloomington Stock Center	BDSC_45016
UAS-Rpr	Bloomington Stock Center	BDSC_5823
Akh-GAL4	Bloomington Stock Center	BDSC_25684
AstA-R2-GAL4	Bloomington Stock Center	BDSC_76727
UAS-srcGFP	Bloomington Stock Center	BDSC_5432
UAS-AstA-i-JF	Bloomington Stock Center	BDSC_25866
UAS-AstA-i	Vienna Drosophila Resource Center	v103215
TrpA1-GAL4	Bloomington Stock Center	BDSC_36922
TrpA1-A-KI-T2A-GAL4	Gift from Drs. Yang Xiang and Pengyu Gu	Previous study [5]
TrpA1-B-KI-T2A-GAL4	Gift from Drs. Yang Xiang and Pengyu Gu	Previous study [5]
TrpA1-C-KI-T2A-GAL4	Gift from Drs. Yang Xiang and Pengyu Gu	Previous study [5]
TrpA1-D-KI-T2A-GAL4	Gift from Drs. Yang Xiang and Pengyu Gu	Previous study [5]
*UAS-AR2-i-*JF	Bloomington Stock Center	BDSC_25935

UAS-AR2-i	National Institute of Genetics, Japan	NIG10001R-1
UAS-TrpA1-A	Bloomington Stock Center	BDSC_26264
UAS-CaLexA-GFP	Bloomington Stock Center	BDSC_66542
Akh^{SAP}	Gift from Dr. Ronald Kühnlein	Previous study [6]
Akh^{A}	Gift from Dr. Ronald Kühnlein	Previous study [6]
w^{1118}	This study	N/A
yv; attp2	This study	N/A
**Antibodies**		
Rabbit anti-AstA	Our previous study	Previous study [7]
Rabbit anti-Tk	Our previous study	Previous study [8]
Rabbit anti-Akh	This study	N/A
Mouse anti-Pros	Development Studies Hybridoma Bank	Prospero (MR1A)

References:

1 Koh, K. *et al.* Identification of SLEEPLESS, a sleep-promoting factor. *Science* **321**, 372-376 (2008).

2 Kume, K., Kume, S., Park, S. K., Hirsh, J. & Jackson, F. R. Dopamine is a regulator of arousal in the fruit fly. *J Neurosci* **25**, 7377-7384 (2005).

3 Vaccaro, A. *et al.* Sleep Loss Can Cause Death through Accumulation of Reactive Oxygen Species in the Gut. *Cell* **181**, 1307-1328 e1315 (2020).

4 Song, W. *et al.* Midgut-Derived Activin Regulates Glucagon-like Action in the Fat Body and Glycemic Control. *Cell Metab* **25**, 386-399 (2017).

5 Gu, P. *et al.* Polymodal Nociception in Drosophila Requires Alternative Splicing of TrpA1. *Curr Biol* **29**, 3961-3973 e3966 (2019).

6 Galikova, M. *et al.* Energy Homeostasis Control in Drosophila Adipokinetic Hormone Mutants. *Genetics* **201**, 665-683 (2015).

7 Veenstra, J. A., Agricola, H. J. & Sellami, A. Regulatory peptides in fruit fly midgut. *Cell Tissue Res* **334**, 499-516 (2008).

8 Song, W., Veenstra, J. A. & Perrimon, N. Control of lipid metabolism by tachykinin in Drosophila. *Cell Rep* **9**, 40-47 (2014).

Supplementary Table S3. Genotypes in this study. Related to each Figure.

**Figure 1**	
**a**	*50A07-LexA>+*<br>*50A07-LexA>LexAop-TrpA1*
**b**	w^{iso31}<br>$sss^{\Delta40}$
**c**	w^{1118} control<br>w^{1118} mechanical sleep deprivation
**d**	*50A07-LexA>+*<br>*50A07-LexA>LexAop-TrpA1*<br>*68A07-LexA>+*<br>*68A07-LexA>LexAop-TrpA1*
**e**	*11H05-GAL4>+*<br>*11H05-GAL4> UAS-TrpA1*
**f**	w^{iso31}<br>$sss^{\Delta40}$
**g**	w^{1118} control<br>w^{1118} mechanical sleep deprivation
**h-i**	*50A07-LexA>+*<br>*50A07-LexA>LexAop-TrpA1*<br>*68A07-LexA>+*<br>*68A07-LexA>LexAop-TrpA1*<br>w^{iso31}<br>$sss^{\Delta40}$<br>w^{1118} control<br>w^{1118} mechanical sleep deprivation

**Figure 2**	
**a**	w^{1118}
**b**	*50A07-LexA>+*<br>*50A07-LexA>LexAop-TrpA1*<br>*68A07-LexA>+*<br>*68A07-LexA>LexAop-TrpA1*<br>w^{iso31}<br>$sss^{\Delta40}$<br>w^{1118} control<br>w^{1118} mechanical sleep deprivation
**c**	*50A07-LexA>+*<br>*50A07-LexA>LexAop-TrpA1*<br>*11H05-GAL4>+*<br>*11H05-GAL4> UAS-TrpA1*<br>w^{iso31}<br>$sss^{\Delta40}$<br>w^{1118} control<br>w^{1118} mechanical sleep deprivation

**d**	*50A07-LexA>+*<br>*50A07-LexA>LexAop-TrpA1*<br>*68A07-LexA>+*<br>*68A07-LexA>LexAop-TrpA1*<br>*11H05-GAL4>+*<br>*11H05-GAL4> UAS-TrpA1*<br>*w*^{iso31}<br>*sss*$^{\Delta 40}$<br>*w*^{1118} control<br>*w*^{1118} mechanical sleep deprivation
**e**	*AstA*^{33}*-GAL4>UAS-srcGFP*
**f**	*AstA*^{33}*-GAL4>UAS-srcGFP*
**g**	*AstA*^{33}*-GAL4>+*<br>*AstA*^{33}*-GAL4>UAS-AstA-i*<br>*pros-GAL4>+*<br>*pros-GAL4>UAS-AstA-i*
**h**	*AstA*^{33}*-GAL4>+*<br>*AstA*^{33}*-GAL4>UAS-AstA-i*

**Figure 3**	
**a**	*AstA*^{33}*-GAL4>+*<br>*AstA*^{33}*-GAL4>UAS-AstA-i*<br>*pros-GAL4>+*<br>*pros-GAL4>UAS-AstA-i*
**b**	*50A07-LexA> LexAop-TrpA1// AstA*^{33}*-GAL4>+*<br>*50A07-LexA>LexAop-TrpA1// AstA*^{33}*-GAL4>UAS-AstA-i*
**c**	*50A07-LexA> LexAop-TrpA1// AstA*^{33}*-GAL4>+*<br>*50A07-LexA>LexAop-TrpA1// AstA*^{33}*-GAL4>UAS-AstA-i*
**d**	*50A07-LexA>+// AstA*^{33}*-GAL4>+*<br>*50A07-LexA>LexAop-TrpA1// AstA*^{33}*-GAL4>+*<br>*50A07-LexA> +// AstA*^{33}*-GAL4> UAS-AstA-i*<br>*50A07-LexA>LexAop-TrpA1// AstA*^{33}*-GAL4>UAS-AstA-i*<br>*50A07-LexA>+// pros-GAL4>+*<br>*50A07-LexA>LexAop-TrpA1// pros-GAL4>+*<br>*50A07-LexA> +// pros-GAL4> UAS-AstA-i*<br>*50A07-LexA>LexAop-TrpA1// pros-GAL4>UAS-AstA-i*
**e**	*50A07-LexA>+// AstA*^{33}*-GAL4>+*<br>*50A07-LexA>LexAop-TrpA1// AstA*^{33}*-GAL4>+*<br>*50A07-LexA>LexAop-TrpA1// AstA*^{33}*-GAL4>UAS-AstA-i-JF*<br>*68A07-LexA>+// AstA*^{33}*-GAL4>+*<br>*68A07-LexA>LexAop-TrpA1// AstA*^{33}*-GAL4>+*<br>*68A07-LexA>LexAop-TrpA1// AstA*^{33}*-GAL4>UAS-AstA-i-JF*
**f**	*AstA*^{33}*-GAL4>+*<br>*AstA*^{33}*-GAL4>UAS-AstA-i*<br>*pros-GAL4>+*<br>*pros-GAL4>UAS-AstA-i*

**g**	*AstA*^{33}*-GAL4>+* control<br>*AstA*^{33}*-GAL4>+* mechanical sleep deprivation<br>*AstA*^{33}*-GAL4>UAS-AstA-I* control<br>*AstA*^{33}*-GAL4>UAS-AstA-I* mechanical sleep deprivation<br>*pros-GAL4>+* control<br>*pros-GAL4>+* mechanical sleep deprivation<br>*pros-GAL4>UAS-AstA-i* control<br>*pros-GAL4>UAS-AstA-i* mechanical sleep deprivation

**Figure 4**	
**a**	*50A07-LexA>+*<br>*50A07-LexA>LexAop-TrpA1*<br>*11H05-GAL4>+*<br>*11H05-GAL4> UAS-TrpA1*<br>*w*^{iso31}<br>*sss*$^{\Delta 40}$
**b**	*50A07-LexA>+*<br>*50A07-LexA>LexAop-TrpA1*<br>*68A07-LexA>+*<br>*68A07-LexA>LexAop-TrpA1*
**c**	*50A07-LexA>+*<br>*50A07-LexA>LexAop-TrpA1*<br>*68A07-LexA>+*<br>*68A07-LexA>LexAop-TrpA1*<br>*w*^{iso31}<br>*sss*$^{\Delta 40}$<br>*11H05-GAL4>+*<br>*11H05-GAL4> UAS-TrpA1*<br>*w*^{1118} control<br>*w*^{1118} mechanical sleep deprivation
**d**	*50A07-LexA>LexAop-TrpA1// AstA*^{33}*-GAL4>+*<br>*50A07-LexA>LexAop-TrpA1// AstA*^{33}*-GAL4>UAS-AstA-i*<br>*68A07-LexA>LexAop-TrpA1// AstA*^{33}*-GAL4>+*<br>*68A07-LexA>LexAop-TrpA1// AstA*^{33}*-GAL4>UAS-AstA-i*
**e**	*50A07-LexA>+*<br>*50A07-LexA>LexAop-TrpA1*<br>*50A07-LexA>+//Akh*$^{A/SAP}$<br>*50A07-LexA>LexAop-TrpA1//Akh*$^{A/SAP}$<br>*68A07-LexA>+*<br>*68A07-LexA>LexAop-TrpA1*<br>*68A07-LexA>LexAop-TrpA1//Akh*$^{A/SAP}$
**f**	*w*^{1118} control<br>*Akh*$^{A/SAP}$ control<br>*w*^{1118} mechanical sleep deprivation<br>*Akh*$^{A/SAP}$ mechanical sleep deprivation

**Figure 5**	
**a**	αTC1+PBS<br>αTC1+Galanin<br>αTC1+Galanin+M35
**b**	*C57BL/6*+PBS<br>*C57BL/6*+Galanin<br>*C57BL/6*+Galanin+M35
**c**	*C57BL/6*+PBS<br>*C57BL/6*+STZ<br>*C57BL/6*+STZ+M35
**d**	None
**e**	*C57BL/6*+control<br>*C57BL/6*+sleep deprivation
**f**	*C57BL/6*+control<br>*C57BL/6*+sleep deprivation<br>*C57BL/6*+sleep deprivation+M35
**g**	*C57BL/6*+control<br>*C57BL/6*+sleep deprivation<br>*C57BL/6*+sleep deprivation+M35
**h**	*C57BL/6*+control<br>*C57BL/6*+sleep deprivation<br>*C57BL/6*+sleep deprivation+M35<br>*C57BL/6*+sleep deprivation+GRA
**i**	*C57BL/6*+control<br>*C57BL/6*+sleep deprivation<br>*C57BL/6*+sleep deprivation+M35<br>*C57BL/6*+sleep deprivation+GRA
**j**	*C57BL/6*<br>*C57BL/6*+sleep deprivation<br>$Gcg^{-/-}$+sleep deprivation
**k**	*C57BL/6*<br>*C57BL/6*+sleep deprivation<br>$Gcg^{-/-}$+sleep deprivation
**l**	*C57BL/6*+control<br>*C57BL/6*+sleep deprivation<br>*C57BL/6*+sleep deprivation+M35<br>$Gcg^{-/-}$+sleep deprivation
**m**	*C57BL/6*+control<br>*C57BL/6*+sleep deprivation<br>*C57BL/6*+sleep deprivation+M35<br>$Gcg^{-/-}$+sleep deprivation

**Figure 6**	
**a**	None
**b**	*50A07-LexA>+*<br>*50A07-LexA>LexAop-TrpA1*

	68A07-LexA>+<br>*68A07-LexA>LexAop-TrpA1*<br>w^{iso31}<br>$sss^{\Delta 40}$
**c**	*50A07-LexA>+*<br>*50A07-LexA>LexAop-TrpA1*<br>*68A07-LexA>+*<br>*68A07-LexA>LexAop-TrpA1*<br>w^{iso31}<br>$sss^{\Delta 40}$
**d**	w^{iso31}<br>w^{iso31}+LA<br>$sss^{\Delta 40}$<br>$sss^{\Delta 40}$+LA
**e**	*50A07-LexA>+*<br>*50A07-LexA>LexAop-TrpA1*<br>*50A07-LexA>LexAop-TrpA1*+LA<br>*68A07-LexA>+*<br>*68A07-LexA>LexAop-TrpA1*<br>*68A07-LexA>LexAop-TrpA1*+LA
**f**	*50A07-LexA>+*<br>*50A07-LexA>LexAop-TrpA1*<br>*50A07-LexA>LexAop-TrpA1*+LA<br>*68A07-LexA>+*<br>*68A07-LexA>LexAop-TrpA1*<br>*68A07-LexA>LexAop-TrpA1*+LA
**g**	w^{iso31}<br>w^{iso31}+LA<br>$sss^{\Delta 40}$<br>$sss^{\Delta 40}$+LA

**Figure 7**	
**a**	None
**b**	*TrpA1-GAL4>UAS-srcGFP*
**c**	*50A07-LexA>LexAop-TrpA1// AstA*^{33}*-GAL4>+*<br>*50A07-LexA>LexAop-TrpA1// AstA*^{33}*-GAL4>UAS-TrpA1-i*<br>*AstA*^{33}*-GAL4>+* mechanical sleep deprivation<br>*AstA*^{33}*-GAL4>UAS-TrpA1-i* mechanical sleep deprivation
**d**	*50A07-LexA>LexAop-TrpA1// AstA*^{33}*-GAL4>+*<br>*50A07-LexA>LexAop-TrpA1// AstA*^{33}*-GAL4>UAS-TrpA1-i*<br>*AstA*^{33}*-GAL4>+* mechanical sleep deprivation<br>*AstA*^{33}*-GAL4>UAS-TrpA1-i* mechanical sleep deprivation
**e**	*50A07-LexA>LexAop-TrpA1// AstA*^{33}*-GAL4>+*<br>*50A07-LexA>LexAop-TrpA1// AstA*^{33}*-GAL4>UAS-TrpA1-i*<br>*AstA*^{33}*-GAL4>+* mechanical sleep deprivation<br>*AstA*^{33}*-GAL4>UAS-TrpA1-i* mechanical sleep deprivation

**f**	*50A07-LexA>LexAop-TrpA1// AstA*^{33}*-GAL4>+*<br>*50A07-LexA>LexAop-TrpA1// AstA*^{33}*-GAL4>+ UAS-TrpA1-i*<br>*50A07-LexA>+// AstA*^{33}*-GAL4>+*<br>*50A07-LexA>LexAop-TrpA1// AstA*^{33}*-GAL4>+*<br>*50A07-LexA>+// AstA*^{33}*-GAL4> UAS-TrpA1-i*<br>*50A07-LexA>LexAop-TrpA1// AstA*^{33}*-GAL4>+ UAS-TrpA1-i*
**g**	*AstA*^{33}*-GAL4>+* mechanical sleep deprivation<br>*AstA*^{33}*-GAL4>UAS-TrpA1-i* mechanical sleep deprivation<br>*AstA*^{33}*-GAL4>+* control<br>*AstA*^{33}*-GAL4>+* mechanical sleep deprivation<br>*AstA*^{33}*-GAL4>UAS-TrpA1-i* mechanical sleep deprivation

**Figure S1**	
**a-e**	*w*^{iso31}<br>*DAT*^{fmn}
**f**	*w*^{1118}<br>*UAS-AstA-i /+*<br>*LexAop-TrpA1/+*<br>*UAS-TrpA1/+*
**g**	*w*^{1118}<br>*UAS-AstA-i /+*
**h**	*w*^{1118}<br>*LexAop-TrpA1/+*<br>*UAS-TrpA1/+*
**i**	*elav-GAL4> +*<br>*elav-GAL4> UAS-AstA-i*
**j**	*elav-GAL4> +*<br>*elav-GAL4> UAS-AstA-i*
**k**	*elav-GAL4> +* control<br>*elav-GAL4> +* mechanical sleep deprivation<br>*elav-GAL4> UAS-AstA-i* control<br>*elav-GAL4> UAS-AstA-i* mechanical sleep deprivation

**Figure S2**	
**a**	*AstA*^{33}*-GAL4> UAS-srcGFP*
**b**	*AstA*^{33}*-GAL4> UAS-srcGFP*
**c**	*pros-GAL4> UAS-srcGFP*
**d**	*pros-GAL4> UAS-srcGFP*

**Figure S3**	
**a**	*50A07-LexA> LexAop-GFP*
**b**	*50A07-LexA> LexAop-GFP*
**c**	*68A07-LexA> LexAop-GFP*
**d**	*11H05 -GAL4> UAS-srcGFP*

**Figure S4**	
**a**	w^{1118} fed<br>w^{1118} starved
**b**	$AstA^{33}$-*GAL4>+* fed<br>$AstA^{33}$-*GAL4>UAS-AstA-i* fed<br>$AstA^{33}$-*GAL4>+* starved<br>$AstA^{33}$-*GAL4>UAS-AstA-i* starved<br>*pros-GAL4>+* fed<br>*pros-GAL4>UAS-AstA-i* fed<br>*pros-GAL4>+* starved<br>*pros-GAL4>UAS-AstA-i* starved
**c**	$AstA^{33}$-*GAL4>+*<br>$AstA^{33}$-*GAL4>UAS-AstA-i JF*
**d**	$AstA^{33}$-*GAL4>+*<br>$AstA^{33}$-*GAL4>UAS-AstA-i JF*
**e-h**	$AstA^{33}$-*GAL4>+*<br>$AstA^{33}$-*GAL4> UAS-Rpr*

**Figure S5**	
**a**	*Akh-GAL4> UAS-srcGFP*
**b**	w^{1118}<br>Akh^{A}<br>Akh^{SAP}
**c**	w^{1118}<br>Akh^{A}
**d**	w^{1118}<br>Akh^{SAP}<br>w^{1118} fed<br>Akh^{SAP} starved
**e**	$AstA^{33}$-*GAL4>+*<br>$AstA^{33}$-*GAL4> UAS-AstA-i*
**f**	$AstA^{33}$-*GAL4>+*<br>$AstA^{33}$-*GAL4> UAS-AstA-i*

**Figure S6**	
**a**	*AstA-R2-GAL4> UAS-srcGFP*
**b**	*Akh-GAL4> UAS-CaLexA-GFP*
**c**	*Akh-GAL4> +*<br>*Akh-GAL4> UAS- AstA-R2-i*
**d**	*Akh-GAL4> +*<br>*Akh-GAL4> UAS- AstA-R2-i-JF*<br>*Akh-GAL4>UAS- AstA-R2-i*
**e**	$AstA^{33}$-*GAL4>+*<br>$AstA^{33}$-*GAL4> UAS-AstA-i*<br>$AstA^{33}$-*GAL4>+// AkhA*$^{A/SAP}$

	AstA^{33}*-GAL4> UAS-AstA-i// AkhA*$^{A/SAP}$
**f-h**	*AstA*^{33}*-GAL4>+// Akh-GAL4> +*<br>*AstA*^{33}*-GAL4> UAS-AstA-i //Akh-GAL4> +*<br>*AstA*^{33}*-GAL4>+// Akh-GAL4>UAS- AstA-R2-i*<br>*AstA*^{33}*-GAL4> UAS-AstA-i // Akh-GAL4>UAS- AstA-R2-i*

**Figure S7**	
**a**	αTC1+PBS<br>αTC1+Galanin<br>αTC1+Galanin+M35
**b**	*C57BL/6*+PBS<br>*C57BL/6*+Galanin<br>*C57BL/6*+Galanin+M35
**c**	*Gcg*$^{-/-}$+ PBS<br>*Gcg*$^{-/-}$+ Galanin
**d**	None
**e**	*C57BL/6*+PBS<br>*C57BL/6*+STZ
**f**	*C57BL/6*+PBS<br>*C57BL/6*+STZ<br>*C57BL/6*+STZ+M35
**g**	*C57BL/6*+PBS<br>*C57BL/6*+STZ<br>*C57BL/6*+STZ+M35
**h-k**	*C57BL/6*<br>*Gcg*$^{-/-}$

**Figure S8**	
**a**	*50A07-LexA>+*<br>*50A07-LexA>LexAop-TrpA1*<br>*50A07-LexA>LexAop-TrpA1*+LA<br>*68A07-LexA>+*<br>*68A07-LexA>LexAop-TrpA1*<br>*68A07-LexA>LexAop-TrpA1*+LA
**b**	*w*^{iso31}<br>*sss*^{Δ40}<br>*sss*^{Δ40}+LA
**c**	*w*^{iso31}<br>*sss*^{Δ40}<br>*sss*^{Δ40}+LA
**d**	*w*^{1118} control<br>*w*^{1118} mechanical sleep deprivation
**e**	*50A07-LexA>+*<br>*50A07-LexA>LexAop-TrpA1*<br>*50A07-LexA>LexAop-TrpA1*+LA<br>*68A07-LexA>+*

	68A07-LexA>LexAop-TrpA1<br>*68A07-LexA>LexAop-TrpA1*+LA
**f**	*TrpA1-A-KI-T2A-GAL4>UAS-srcGFP*<br>*TrpA1-B-KI-T2A-GAL4>UAS-srcGFP*<br>*TrpA1-C-KI-T2A-GAL4>UAS-srcGFP*<br>*TrpA1-D-KI-T2A-GAL4>UAS-srcGFP*

Supplementary Table S4. Oligonucleotide information.

**Oligonucleotides**
Fly *RpL32*-F: GCTAAGCTGTCGCACAAATG
Fly *RpL32*-R: GTTCGATCCGTAACCGATGT
Fly *AstA*-F*:* CAAGAGGTCTCGTCCCTACT
Fly *AstA*-R: CTTGTTCTGTCGGCCAGGTC
Fly *tobi*-F: GTCATGCATCCTGTGTGGTC
Fly *tobi*-R*:* GATTTCCAGCTGGCTGTTGT
Fly *Akh*-F: TCCCAAGAGCGAAGTCCTCA
Fly *Akh*-R: CCAGAAAGAGCTGTGCCTGA
Fly *AkhR*-F: GCTATCCACGGACCTGATGTG
Fly *AkhR*-R: CTGTCGAGCGATATGCAGACC
Mouse *β-actin*-F: AGTGTGACGTTGACATCCGTA
Mouse *β-actin*-R: GCCAGAGCAGTAATCTCCTTCT
Mouse *Gcg*-F: GATCATCCCAGCTTCCCAG
Mouse *Gcg*-R: CTGGTAAAGGTCCCTTCAGC
Mouse *Gal*-F: GGCAGCGTTATCCTGCTAGG
Mouse *Gal*-R: CTGTTCAGGGTCCAACCTCT
Mouse *GalR1*-F: GCCGCGATGTCTGTGGATCG
Mouse *GalR1*-R: CGATGGACAGCGCCCAGATG
Mouse *GalR2*-F: GTGTGCCACCCAGCGTGGAG
Mouse *GalR2*-R: TGGTGCGCGCATAGGTCAGG
Mouse *GalR3*-F: CCTGCCTCAACCCGCTCGTC
Mouse *GalR3*-R: TGAAGGCGGTGGTGGTGGTG
Mouse *G6p*-F: CCGGTGTTTGAACGTCATCT
Mouse *G6p*-R: CAATGCCTGACAAGACTCCA
